# Network-specific metabolic and haemodynamic effects elicited by non-invasive brain stimulation

**DOI:** 10.1038/s44220-023-00046-8

**Published:** 2023-05-01

**Authors:** Mark C. Eldaief, Stephanie McMains, David Izquierdo-Garcia, Mohammad Daneshzand, Aapo Nummenmaa, Rodrigo M. Braga

**Affiliations:** 1 Department of Neurology, Massachusetts General Hospital, Harvard Medical School, Boston, MA, USA.; 2 Department of Psychiatry, Massachusetts General Hospital, Harvard Medical School, Boston, MA, USA.; 3 Center for Brain Science, Neuroimaging Facility, Harvard University, Cambridge, MA, USA.; 4 Athinoula A. Martinos Center for Biomedical Imaging, Massachusetts General Hospital, Charlestown, MA, USA.; 5 Cognitive Neuroimaging Center, Boston University, Boston, MA, USA.; 6 Department of Neurology, Northwestern University, Feinberg School of Medicine, Chicago, IL, USA.

## Abstract

Repetitive transcranial magnetic stimulation (TMS), when applied to the dorsolateral prefrontal cortex (dlPFC), treats depression. Therapeutic effects are hypothesized to arise from propagation of local dlPFC stimulation effects across distributed networks; however, the mechanisms of this remain unresolved. dlPFC contains representations of different networks. As such, dlPFC TMS may exert different effects depending on the network being stimulated. Here, to test this, we applied high-frequency TMS to two nearby dlPFC targets functionally embedded in distinct anti-correlated networks—the default and salience networks— in the same individuals in separate sessions. Local and distributed TMS effects were measured with combined ^18^fluorodeoxyglucose positron emission tomography and functional magnetic resonance imaging. Identical TMS patterns caused opposing effects on local glucose metabolism: metabolism increased at the salience target following salience TMS but decreased at the default target following default TMS. At the distributed level, both conditions increased functional connectivity between the default and salience networks, with this effect being dramatically larger following default TMS. Metabolic and haemodynamic effects were also linked: across subjects, the magnitude of local metabolic changes correlated with the degree of functional connectivity changes. These results suggest that TMS effects upon dlPFC are network specific. They also invoke putative antidepressant mechanisms of TMS: network de-coupling.

Repetitive transcranial magnetic stimulation (TMS), when applied to the dorsolateral prefrontal cortex (dlPFC), has established efficacy as a treatment for major depressive disorder (MDD)^1^. Therapeutic TMS protocols used to treat depression are either applied in an ‘excitatory’ (for example, with high-frequency (≥5 Hz) or intermittent theta burst stimulation) or in an ‘inhibitory’ (for example, with low-frequency (1 Hz) or continuous theta burst stimulation) pattern,^2,3^ and can be applied once daily for several weeks^4^ or in an accelerated fashion as multiple daily sessions (for example, in the Stanford Neuromodulation Therapy protocol^5^). Across protocols, the effects of TMS are thought to result from local changes in neural activity and metabolism at the dlPFC stimulation site, which propagate trans-synaptically across distributed corticolimbic circuits, including the subgenual cingulate (sgACC)^6,7^. There is mounting evidence that TMS exerts its anti-depressant effects through circuit mechanisms^8–10^. For example, the strength of connectivity between the sgACC and the dlPFC target predicts clinical efficacy^10–13^. Still, the precise local and distributed mechanisms underlying TMS neuromodulation in MDD are not resolved. A deeper mechanistic understanding of how local effects of dlPFC stimulation lead to distributed changes in brain activity would have important clinical implications. For instance, clinical response rates of TMS in MDD are variable, with up to half of patients failing to benefit^14^. Thus, understanding the possible basis of this heterogeneity could improve clinical efficacy.

Local effects of TMS are widely assumed to be pattern specific, and this is supported by extant evidence. For example, high-frequency TMS appears to increase local cortical activity, whereas low-frequency TMS has the opposite effect^15,16^. However, most studies examining the local effects of TMS have been performed in motor cortex, where the local impact of TMS is measured with downstream recordings of the motor evoked potential at affected muscles^17^. While some combined TMS and positron emission tomography (PET) studies in dlPFC (and other association cortex regions) have echoed these frequency-specific findings—that is, with high-frequency TMS increasing and low-frequency TMS decreasing local metabolism and local regional cerebral blood flow (rCBF)^18–21^—other studies have produced conflicting results. For example, Eisenegger et al. demonstrated local increases in target rCBF following low-frequency TMS to the right dlPFC^22^. Also, Knoch et al. showed that both high- and low-frequency TMS can increase rCBF at a dlPFC target^23^.

Beyond local effects, it is imperative to assess how changes at the cortical target are parlayed into distributed changes. Studies support long-range cortico-cortical effects of TMS. For example, interleaved TMS/functional magnetic resonance imaging (fMRI), where TMS is administered within the scanner between image acquisitions, has revealed transient increases under the TMS coil that spread to anatomically connected regions^24–26^. However, studies showing network-specific TMS changes have mostly targeted sensorimotor areas, with conflicting results encountered when stimulation is applied to association areas. For example, while our work has suggested that long-range changes from stimulating association areas occur largely within distributed regions that are functionally connected to the stimulation site^27,28^, that is, within the stimulated network, other studies have found functional changes that are not network selective^29,30^.

One possible reason for these inconsistent findings is that the effects of TMS may vary depending on which network is stimulated. The dlPFC is large and functionally heterogeneous, comprising multiple juxtaposed subregions that are embedded in distinct, large-scale distributed association networks^31^. Different studies may have variably stimulated different networks, even when TMS has been targeted to the dlPFC in each case. These networks, which can be distinguished on the basis of slow intrinsic correlations of the blood oxygenation level-dependent (BOLD) signal^32–34^, may also operate at distinct electrophysiological frequencies^35–39^, and as such may respond differently to the same stimulation frequency. This raises the interesting hypothesis that network identity, or ‘functional architecture’, at the dlPFC stimulation site is an important factor in determining local and distributed TMS effects. If true, precise targeting of functional regions within the dlPFC would be necessary to produce reliable and intended effects of TMS, both in clinical and in investigational contexts.

In this Article, to test this hypothesis, we applied identical patterns of stimulation to two distinct dlPFC regions in the same participants and measured the effects upon metabolism and network coupling. Metabolism and BOLD functional connectivity (FC) were estimated using combined ^18^F-fuorodeoxyglucose-PET (FDG-PET) and fMRI. Twenty individuals took part in a within-subject cross-over counter-balanced design, in which each subject attended three visits on separate days (Fig. 1). Of these twenty, data from 16 subjects were carried through to the final analysis. During their first (baseline) visit, subjects underwent hybrid FDG-PET–fMRI imaging. This provided resting-state BOLD data, which was used for FC definition of stimulation sites, as well as a baseline measure of whole-brain glucose metabolism. As FC with the sgACC has been linked to the anti-depressant efficacy of TMS^11,13,40,41^, we selected two dlPFC targets that were functionally coupled to the sgACC in each individual (‘Definition of TMS targets’ in Methods). One target was selected as the region showing maximal positive FC with the sgACC, while the other target showed maximal negative FC with this region. Through this procedure, our stimulation targeted two anti-correlated regions that belonged to anti-correlated distributed networks:^42^ Positively correlated targets fell within the default network (DN), and negatively correlated targets fell within the salience network (SAL) (Fig. 2 and Supplementary Fig. 1).

Subjects then returned for two further FDG-PET–fMRI visits during which TMS was administered. High-frequency (20 Hz) TMS was administered with identical patterns at one of the dlPFC targets per visit. TMS was delivered with online neuronavigation to ensure that the individually derived targets were stimulated accurately (with an anticipated error term of a few mm^43^). To test the immediate haemodynamic effects of TMS, an fMRI-only session was collected immediately before TMS, and a hybrid FDG-PET–fMRI session (on the same scanner) was collected immediately after TMS. This replicated our offline protocol design^27,28^.

On the basis of the substantial literature involving TMS to motor cortex^15,18^, we predicted that 20 Hz TMS would increase target metabolism at both targets, that is, TMS to a given target would increase metabolism at the stimulated target and not at the non-stimulated target. With respect to FC changes, we predicted that 20 Hz TMS would decrease FC in the stimulated network. We based this prediction on our own work^27^, as well as on results from other studies examining the effects of ‘excitatory’ repetitive TMS upon within-network FC^44–46^. We also predicted FC changes occurring between networks. We based this prediction on a recent meta-analysis showing that extra-network effects of TMS are common^47^, and on specific studies demonstrating inter-network effects of stimulating dlPFC^48,49^. Lastly, we hypothesized that the degree of change in metabolism at the target would correlate to the degree of FC changes within the distributed network, indicating a direct relationship. That is, across subjects, local metabolic changes at the stimulation site would predict long-range changes in FC.

## Results

### Confirmation of network identity at target sites

Figure 2 outlines the stimulation target selection procedure. In each subject, two dlPFC stimulation targets were defined on the basis of maximal positive and maximal negative FC with a seed-based FC map (Fig. 2) derived by Fox et al. (*n* = 98) (ref. 40) (see ‘Definition of TMS targets’). To confirm the network identity of the stimulation targets defined in our cohort, the same map from Fox et al. was used to compute seed-based FC maps in all 16 subjects. Figure 2c shows the target locations superimposed on the resulting group-averaged FC map. The targets were predominantly located within distinct dlPFC regions that belonged to two broad networks—one showing positive (yellow–red regions in Fig. 2c) and one showing negative correlation (blue regions) with the FC seed map. By visual inspection, the anatomical distribution of these correlation maps resembled the DNs and SALs. To confirm these network identities, we superimposed the target sites on the Yeo et al.^32^ parcellation (Fig. 2d). The positively correlated targets were predominantly located within the boundaries of the group-defined DN and were thus labelled ‘DN’ targets. In contrast, the negatively correlated sites were predominantly located at or near the SAL and were thus labelled ‘SAL’ targets. In the Yeo parcellation, some of the SAL targets fell outside the small SAL network region in the dlPFC and were probably located within regions belonging to the frontoparietal control network (FPN). In addition to the Yeo parcellation, we also visually examined the overlap between the group-averaged FC maps (minimum *z* value 0.2) using whole-network regions of interest (ROIs) of the DN and SAL and the respective derived targets. Twelve out of sixteen derived SAL targets overlapped with the group-defined SAL, and all 16 DN targets overlapped with the group-defined DN network. Importantly, in all cases, targets were defined using each individual’s FC maps, hence the lack of alignment with the group-averaged network could be a consequence of true inter-individual differences in functional anatomy. To further confirm that the target sites were within the default and SALs, we next correlated the average BOLD time course from a sphere centred at each target with the average time courses within each of the whole-brain network ROIs encompassing each of the seven major cortical networks from Yeo et al.^32^. The SAL targets showed highest correlation with the SAL ROI (mean *z*-transformed *r* value 0.28, standard deviation (s.d.) 0.15) and the DN targets showed highest correlation with the DN ROI (mean *z*-transformed *r* value 0.34, s.d. 0.17). As a final confirmation, we used each individual’s target site as the seed and averaged the resulting FC maps across subjects. The resultant FC map recapitulated the default and SALs, respectively (Supplementary Fig. 1). Thus, in each subject, two dlPFC stimulation targets were defined that were located within distinct functional regions that were determined, through several means, to belong to two different and anti-correlated distributed networks.

### TMS at 20 Hz leads to opposing effects on glucose metabolism

**Fig. 1 |:**
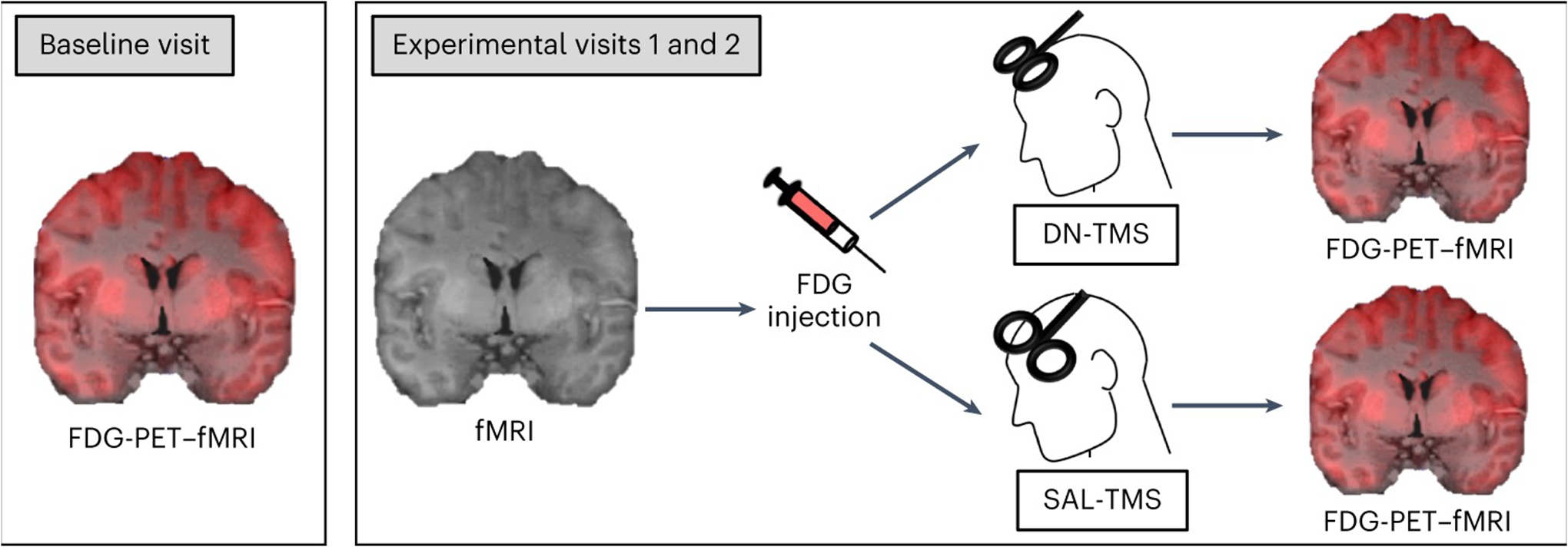
Experimental design. Subjects participated in three visits on separate days: a baseline visit and two experimental visits. During their baseline visit (left), participants underwent simultaneous FDG-PET–fMRI. Data from this baseline visit were used to define two targets for repetitive TMS within each individual, on the basis of FC estimates. One stimulation site targeted the DN and the other targeted the SAL. Target site locations are shown in Fig. 2. Subjects then returned for two experimental visits on separate days. During the experimental visits, subjects first underwent an fMRI scan, after which they were administered an FDG injection a few minutes before high-frequency (20 Hz) repetitive TMS to one of the target sites, in a cross-over design. Following about 22.5 min of repetitive TMS, subjects were immediately scanned using simultaneous FDG-PET–fMRI. The two experimental visits only differed with respect to the target site of stimulation (DN-TMS versus SAL-TMS) and the target order was counter-balanced across subjects.

Sessions where stimulation was applied to the SAL target sites are hereafter referred to as ‘SAL-TMS’, with ‘DN-TMS’ denoting sessions where the DN targets were stimulated. Whole-brain normalized standardized uptake values (WBn-suv) were compared between stimulation sessions and the initial baseline visit (Fig. 1) to quantify the effects of stimulation on cerebral metabolism. Figure 3 shows the effects of SAL-TMS and DN-TMS on local metabolism at the target site. Local WBn-suv increased significantly at the SAL target locations following high-frequency (20 Hz) stimulation to this region (SAL-TMS > baseline; average ΔWBn-suv 0.054, *P* = 0.008, Cohen’s *d* = 0.58; Fig. 3 and Supplementary Fig. 2). Contrary to our expectations, local WBn-suv values decreased significantly at the DN target following 20 Hz TMS to this region (DN-TMS > baseline, average ΔWBn-suv −0.06, *P* = 0.006, Cohen’s *d* = 0.54; Fig. 3). Thus, whereas we observed the expected increase in glucose metabolism following high-frequency SAL-TMS, we did not observe the anticipated increases in metabolism at the DN target following equivalent DN-TMS. Visual analysis of individual-level data suggested that there were considerable differences across subjects in effects elicited at both target sites, which we include for transparency in Supplementary Fig. 2. Direct comparison between the conditions further supported these divergent effects (at SAL targets, SAL-TMS > DN-TMS, average ΔWBn-suv 0.05, *P* = 0.01; at DN targets, DN-TMS > SAL-TMS, average ΔWBn-suv −0.08, *P* = 0.02). Moreover, the non-stimulated target did not change its glucose metabolism following TMS at the other site. That is, metabolism at the SAL target did not significantly change following DN-TMS as compared with baseline (ΔWBn-suv −0.001, *P* = 0.95). Similarly, metabolism at the DN target did not significantly change following SAL-TMS as compared with baseline (ΔWBn-suv 0.01, *P* = 0.44). To explore potential factors explaining these opposing effects, we visually examined the cortical depth and orientation at the DN and SAL targets in each individual, as shown in Supplementary Fig. 3. This did not reveal any systematic differences in depth or sulcal morphology at the site of stimulation that might have explained the results.

#### Network-level metabolic changes.

Given that the two targets were functionally located in distinct networks, we next assessed whether TMS changed whole-brain metabolism in a topographically distinct manner across stimulation targets. We first conducted a network-level analysis in which we quantified FDG metabolism within each of the seven networks. More specifically, we assessed FDG metabolism changes in whole-network ROIs derived from the Yeo et al.^32^ parcellation. To specifically probe network-level effects, we excluded metabolism changes at the respective targets, for example, in the case of SAL-TMS, the SAL ROI used excluded the SAL targets, and in the case of DN-TMS, the DN ROI used excluded the DN targets. The SAL was the only network showing significantly increased metabolism after stimulation of both the SAL and DN targets (bar charts in Fig. 4). In a direct comparison, significantly higher increases in glucose metabolism were observed within the SAL following SAL-TMS compared with DN-TMS. We next mapped voxel-wise metabolic changes to assess the spatial distribution of effects. Figure 4 displays maps showing the change in FDG metabolism following stimulation. SAL-TMS strongly increased metabolism in lateral regions that broadly overlapped with the SAL in both hemispheres (black border outlines in Fig. 4). The effects were most prominent in the contralateral (right) hemisphere; however, the ipsilateral hemisphere also showed evidence of correspondence (for example, see the left frontoinsular cortex in Fig. 4). The correspondence was not perfect, and we also observed evidence of non-specificity, with regions outside the SAL on the right hemisphere also showing metabolic increases. Following the observation of local decreased metabolism at DN target sites following DN-TMS (Fig. 3), we expected to also see distributed decreased metabolism that overlapped with the DN. Instead, DN-TMS led to broad and distributed increases in glucose metabolism, particularly on the contralateral surface. These increases covered a large swathe of the lateral surface, including parts of the inferior parietal region of the SAL, but also extending further along regions of the lateral temporal cortex that overlapped with the DN (Fig. 4). A direct comparison of SAL-TMS and DN-TMS conditions supported that SAL-TMS led to more robust increases in glucose metabolism within the SAL, with a prominent region in the right temporoparietal junction showing close overlap with the SAL (Fig. 4). There were no significant changes in metabolism of the sgACC proper following either stimulation condition: ΔWBn-suv sgACC (SAL-TMS > baseline) −0.004, *P* = 0.70; ΔWBn-suv sgACC (DN-TMS > baseline) 0.01, *P* = 0.26.

### TMS-induced changes in FC

#### Changes in FC following SAL-TMS.

**Fig. 2 |:**
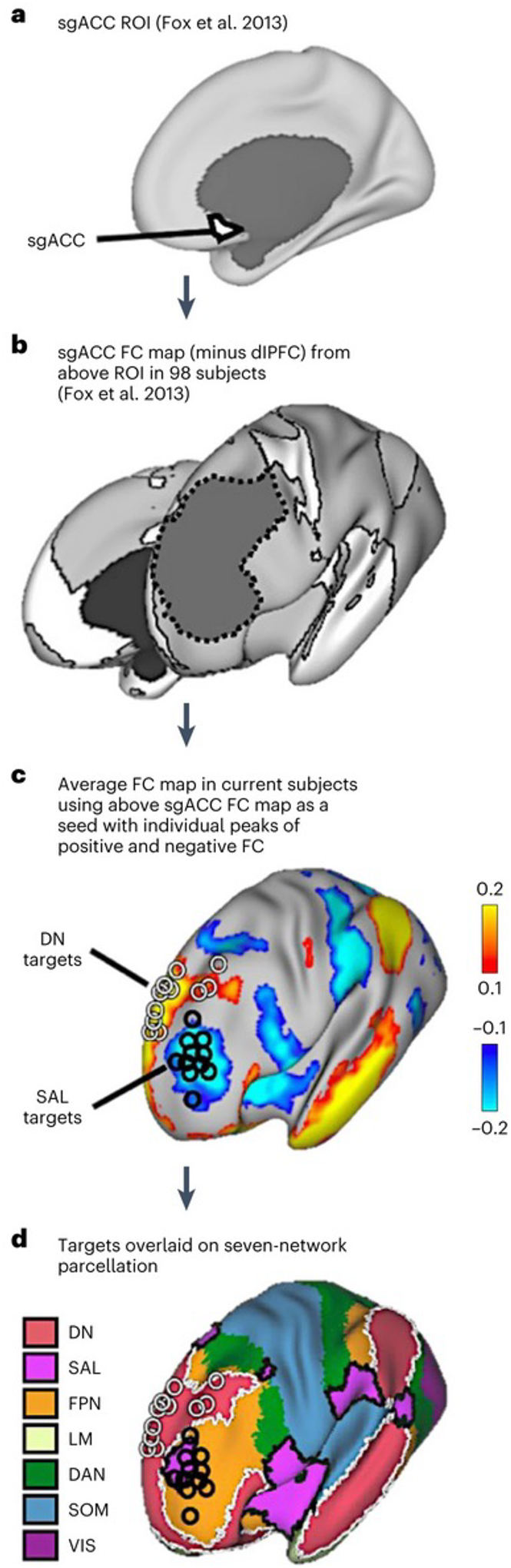
Definition of individualized stimulation targets using FC of the sgACC. **a**,**b**, An sgACC region of interest (**a**) (MNI coordinates: 6, 16 and −10) was used to generate a group-averaged seed-based FC map (**b**), shown in white, in a separate cohort of 98 subjects by Fox et al.^40^. This map excluded the dlPFC (dotted outline) so as to not bias targets being defined there. The map from **b** was itself used as a seed to derive an FC map in each of the subjects in this study. **c**, The resulting group-averaged FC map. This approach allowed us to estimate the FC of the dlPFC with the sgACC robustly, given that the sgACC can suffer from poor signal quality. We next calculated FC strength between the sgACC FC map from (**b**) and 163 regions in the dlPFC in each subject. In each subject, the regions with the most positive and the most negative correlation value with the sgACC FC map were selected as the two stimulation targets for that subject. These targets are shown as black and white rings for each subject in **c**. White rings indicate positively correlated targets and black rings indicate negatively correlated targets. **d**, The network identity of the target sites was determined by overlaying them onto the seven-network parcellation in Yeo et al.^32^. The purple regions with black borders correspond to the SAL and the salmon coloured regions with white borders correspond to the DN. These borders are maintained as landmarks in later figures. The TMS stimulation targets (rings) are overlaid to show that positively correlated targets were predominantly located within the group-defined DN and were thus labelled ‘DN’ targets. The negatively correlated sites were predominantly at or near the SAL and were thus labelled ‘SAL’ targets. Importantly, in all cases, targets were defined using each individual’s FC maps; hence, the lack of alignment with the group-defined network may be due to true inter-individual differences in topography. LM, limbic network; SOM, somatomotor network; VIS, visual network.

We next asked whether stimulation affects FC differently when applied to different networks. Figure 5 shows FC changes in fMRI data as a result of SAL-TMS. Quantitatively, we estimated FC between individual regions covering the whole brain using a region-by-region analysis. Pair-wise correlations of the mean signal within all regions from the Yeo et al.^32^ parcellation (*n* = 51 regions) were calculated. The matrices in Fig. 5a show these correlation values immediately before (pre-TMS) and after (post-TMS) SAL stimulation, as well as their direct comparison. Stimulation of the SAL did not result in significant within-network changes. However, a cluster of DN regions did show modest FC increases (that is, reduced anti-correlation) with SAL regions. This can also be appreciated in Fig. 5b: FC between the mean signal in the ROI encompassing the entire SAL and an ROI encompassing the entire DN was significantly increased following SAL-TMS, and also increased (but not significantly) in the comparison of baseline with post-TMS. We next examined FC changes on voxel-wise FC maps (Fig. 5c). First, the individualized SAL target regions in each subject were used as seeds to create whole-brain FC maps. These maps were then averaged across all subjects to produce a single group-averaged FC map. We observed negligible changes in the FC of the SAL target following SAL-TMS (Fig. 5c, first row). We next tested whether TMS induced changes in the FC of the sgACC region proper (not the sgACC FC map) that was used as the basis of network definition (Fig. 2). FC changes between the sgACC and the DNs and SALs were also negligible following SAL-TMS (Fig. 5c, second row). As these analyses used seeds with a small number of voxels (which may be insufficient for stable signal estimation), we also examined FC changes from ROIs encompassing the entire DNs and SALs to determine whether the whole-brain FC of these large-scale networks was altered by the stimulation. FC changes from SAL-TMS were again modest, but small regions showing increased FC were evident within the boundaries of the DN (Fig. 5c, third row).

#### Changes in FC following DN-TMS.

We next assessed FC changes following DN stimulation using the same approach (Fig. 6). The matrices in Fig. 6a show modest FC decreases within network, and dramatic FC increases (weakened anti-correlations) between regions of the DN and SAL following DN-TMS. On voxel-wise maps, DN-TMS led to subtle decreases in FC between the DN target and the remainder of the DN (blue regions in Fig. 6c, first row), and small but clearer regions of increased FC between the DN target and the SAL (red–yellow regions in Fig. 6c, first row). As the DN targets showed strong positive correlation with the DN and strong negative correlation with the SAL at baseline (Supplementary Fig. 1), these TMS effects can be interpreted as decreased positive correlation of the DN target with the DN and decreased negative correlation with the SAL. Similar results were observed when the sgACC was used as a seed (Fig. 6c, second row). Using larger ROIs encompassing the entire DNs and SALs, we observed subtle FC decreases within each network (blue regions, Fig. 6c, third and fourth rows) and robust FC increases between the two networks (red–yellow regions, Fig. 6c, third and fourth rows). Thus, DN-TMS decreased within-network FC in the DN and robustly increased FC between the DN and SALs. Quantitatively, the FC change between the DN and SAL whole-network ROIs was *z* = 0.10, *P* = 0.009 and Cohen’s *d* = 0.64.

Notably, for both conditions, these effects were quite specific, falling almost exclusively within regions of the DNs and SALs. For instance, consistent FC changes between regions of the other networks were not observed following either SAL-TMS or DN-TMS (Figs. 5 and 6). More specifically, despite the fact that the FPN is robustly represented in dlPFC^32,33,50,51^, and in spite of evidence that dlPFC TMS affects FC between the FPN and the DN^52^, FC changes within the FPN, and between the FPN and other networks, were not observed in either stimulation condition. Similarly, FC changes were not observed between the DN and the dorsal attention network (which are anti-correlated at baseline)^42^.

#### Comparison of pre-/post-TMS, and baseline/post-TMS fMRI data.

For both DN and SAL TMS, we observed differences in the magnitude of effects depending on which data were used as the subtraction condition. For both conditions, the cross-network FC increases reached significance when the pre-TMS fMRI data were used as the subtraction, whereas with the baseline fMRI data we only observed a trend towards significance. To test the robustness of the results, we compared the two approaches directly. Supplementary Fig. 4 shows the resulting FC voxel-wise maps when the two baselines were used for comparison. For DN-TMS, very similar network-specific FC changes were observed regardless of the subtracted condition, supporting that FC changes were induced by TMS, as opposed to being a consequence of the specific comparison data that were used. For SAL-TMS, the effects were notably more robust when the baseline was used as the subtraction.

### Correlation between target metabolism changes and FC changes

**Fig. 3 |:**
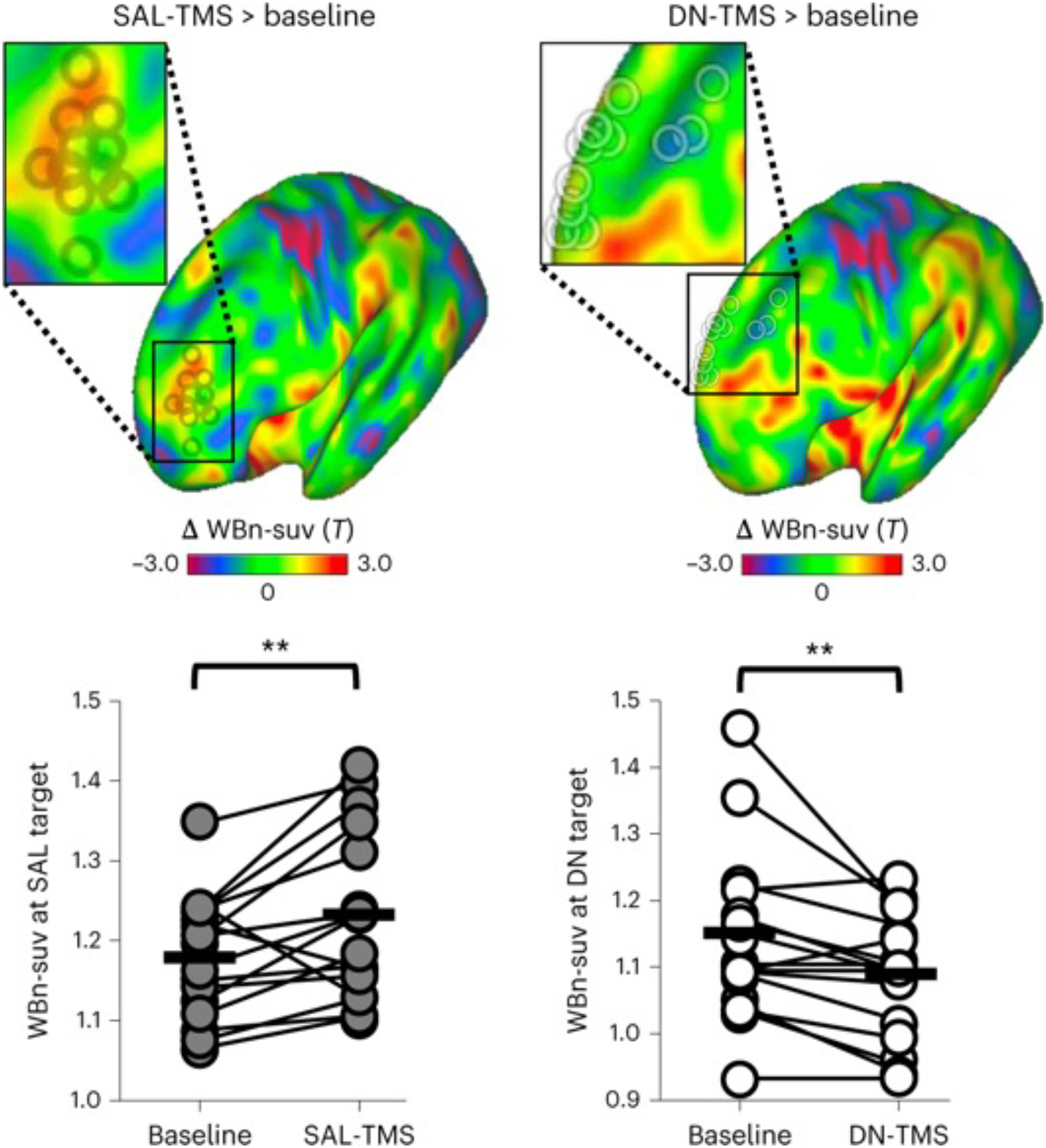
Identical patterns of repetitive TMS at two nearby dorsolateral prefrontal sites lead to opposite effects on local glucose metabolism. Top: surface-rendered unthresholded voxel-wise statistical *T* maps showing the comparison between FDG-PET metabolism after SAL-TMS versus baseline (left) and after DN-TMS versus baseline (right) in the 16 study subjects. Individual target sites are shown in insets as transparent black (SAL targets) and white (DN targets) rings. Increased metabolism is represented as red–yellow and decreased metabolism as blue–maroon. SAL-TMS significantly increased target metabolism and DN-TMS significantly decreased target metabolism. Bottom: graphs showing subject-level (*n* = 16) changes in WBn-suv values at baseline and after TMS (with dark circles indicating changes from SAL-TMS (left) and white circles indicating changes from DN-TMS (right). Thick black horizontal lines represent mean values. Statistical testing involved a paired two-sided *t*-test uncorrected for multiple comparisons: for SAL-TMS > baseline, *P* = 0.008; fFor DN-TMS > baseline *P* = 0.006. **P < 0.01.

Stimulation of the SAL targets led to increased local metabolism at the stimulation site (Fig. 3). If TMS affects both cerebral metabolism and FC, then a logical hypothesis would be that the degree of change in local glucose metabolism following TMS would predict the degree of change in distributed FC—that is, that the two effects share a common mechanism. Across all subjects, we tested whether individual differences in local target metabolism following SAL-TMS predicted individual differences in the FC changes following SAL-TMS. In other words, would individuals who displayed higher increases in SAL target metabolism following SAL-TMS exhibit greater increases in FC (positive correlations), or greater decreases in FC (negative correlations)? Correlation maps between metabolic and haemodynamic changes following SAL-TMS are shown in Supplementary Fig. 5. Following SAL-TMS, correlations between changes in SAL target metabolism and SAL target FC were negligible (Supplementary Fig. 5, top row). However, we observed strong correlations between changes in SAL target metabolism and changes in FC between the sgACC and the SAL (Supplementary Fig. 5, middle left panel). That is, increased SAL target metabolism strongly correlated with increased FC between the sgACC and the SAL. Quantitatively, the correlation between changes in SAL target metabolism and changes in FC between a seed restricted to the sgACC and a seed encompassing the whole SAL map were as follows: *r* = 0.71, *R*^2^ = 0.50, *P* = 0.002). Similarly, correlation maps showed that increased SAL target metabolism was associated with decreased FC between the sgACC and the DN (Supplementary Fig. 5, middle right panel), although quantitatively this only trended towards statistical significance (*r* = −0.48, *R*^2^ = 0.23, *P* = 0.06). Increases in SAL target metabolism also positively correlated with increases in FC between seeds encompassing the entire DNs and SALs (*r* = 0.67, *R*^2^ = 0.45, *P* = 0.004) (Supplementary Fig. 5, bottom left panel).

Stimulation of the DN targets led to decreased local metabolism at the stimulation site (Fig. 3). Thus, we next examined whether, across all subjects, individuals who displayed greater decreases in DN target metabolism following DN TMS exhibited greater decreases in FC (positive correlations), or greater increases in FC (negative correlations; Supplementary Fig. 6). We found that individuals who displayed greater decreases in DN target metabolism also displayed greater increases in FC between the DN target and SAL regions (Supplementary Fig. 6, top right panel). However, this did not reach statistical significance on quantitative measures (*r* = −0.46, *R*^2^ = 0.21, *P* = 0.08). Topographically, greater decreases in DN target metabolism appeared to correlate with decreased FC between the sgACC and the DN (Supplementary Fig. 6, middle left panel), but this did not reach statistical significance (*r* = 0.39, *R*^2^ = 0.15, *P* = 0.13). When the whole DN network map was used as a seed, decreased DN target metabolism was not associated with increased FC between the entire SAL and DN ROIs (*r* = −0.19, *R*^2^ = 0.04, *P* = 0.48). We also performed a subanalysis showing that, for both TMS conditions, the five individuals who exhibited target metabolism changes that diverged the most in each direction from the group trend were more likely to exhibit FC changes that also diverged from the group mean. For example, following SAL-TMS, the five subjects with the lowest increases in metabolism at the SAL target demonstrated very little evidence of FC increases between the SAL and DNs, while the five subjects with the greatest increases at the SAL target demonstrated clear FC increases between the two networks. Similarly, following DN-TMS, the five subjects who did not exhibit metabolic decreases at the DN target also exhibited weak FC increases between the DN and SALs. In contrast, the five individuals exhibiting the most robust decreases at the DN target following DN-TMS showed robust FC increases between the two networks. (Supplemental Fig. 7). Taken together, these findings suggest a degree of regional specificity in the association between TMS-induced metabolic and FC changes.

## Discussion

**Fig. 4 |:**
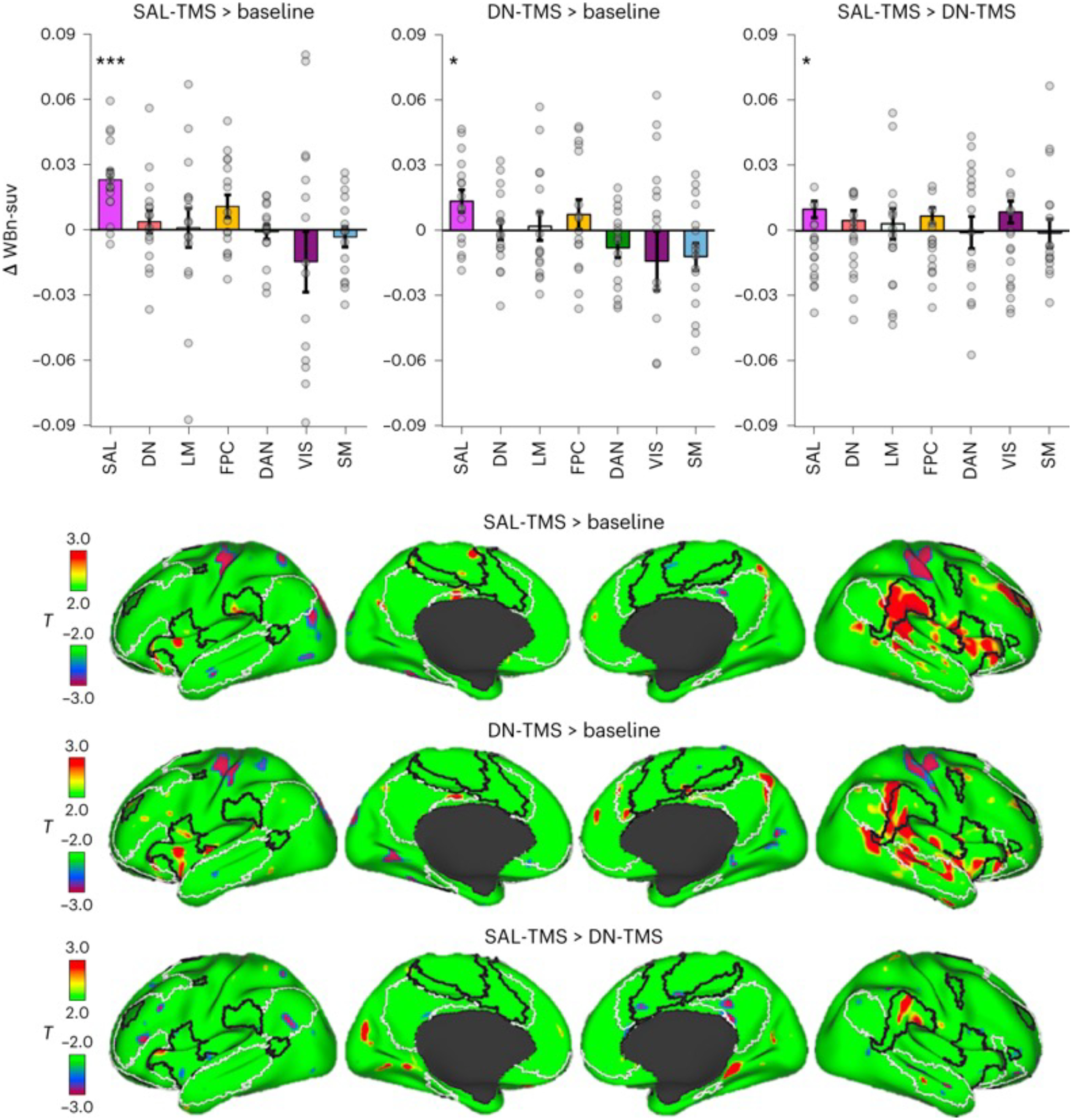
Repetitive TMS leads to distributed effects on glucose metabolism. Top: bar graphs showing metabolic changes in seven major networks from Yeo et al.^32^ following stimulation compared with baseline (SAL-TMS > baseline and DN-TMS > baseline). For these comparisons, metabolic changes at the respective targets were excluded to only show distributed network effects. A direct comparison (SAL-TMS > DN-TMS) is also shown. To illustrate the distribution of changes across subjects, changes for each subject (16 in total) are shown as grey dots. At the network level, both SAL-TMS and DN-TMS increased metabolism significantly within the SAL, but the effect was significantly larger after SAL-TMS. Statistical testing involved a paired two-sided *t*-test uncorrected for multiple comparisons: for SAL-TMS > baseline, *P* = 0.0001 for changes in the SAL. Changes in all other networks were non-significant. For DN-TMS > baseline, *P* = 0.02 for changes in the SAL. Changes in all other networks were non-significant. *P < 0.05, ***P < 0.001. Error bars represent ±standard error of the mean. Bottom: surface-rendered, voxel-wise statistical *T* maps are displayed to show regional changes in metabolism following stimulation (*P* < 0.05, uncorrected). Black outlines demarcate the SAL and white outlines demarcate the DN from Yeo et al.^32^. Stimulation of both SAL and DN target sites led to distributed changes in metabolism; however, the results differed between sites in magnitude and spatial distribution. SAL-TMS was associated with marked increases in metabolism in regions that mostly overlapped with the SAL. The effects were considerably larger on the right hemisphere, contralateral to the site of stimulation. Note in particular the area of strong increase in metabolism within the SAL region in the right temporoparietal junction, as well as the right anterior insula. DN-TMS also led to metabolic changes in multiple distributed regions in the right hemisphere, but we did not see a pattern of network-wide decreased metabolism, as might have been expected due to the metabolic decreases at the DN stimulation targets following DN-TMS. The comparison of SAL-TMS and DN-TMS is shown in the bottom row (red–yellow colours indicate regions with greater metabolic changes after SAL-TMS and blue–maroon indicates regions with greater metabolic changes after DN-TMS). SAL-TMS more robustly increased salience metabolism, particularly in the right temporoparietal junction SAL region. DN-TMS mildly increased metabolism in posterior midline regions, for example, the right posterior cingulate cortex node of the DN.

**Fig. 5 |:**
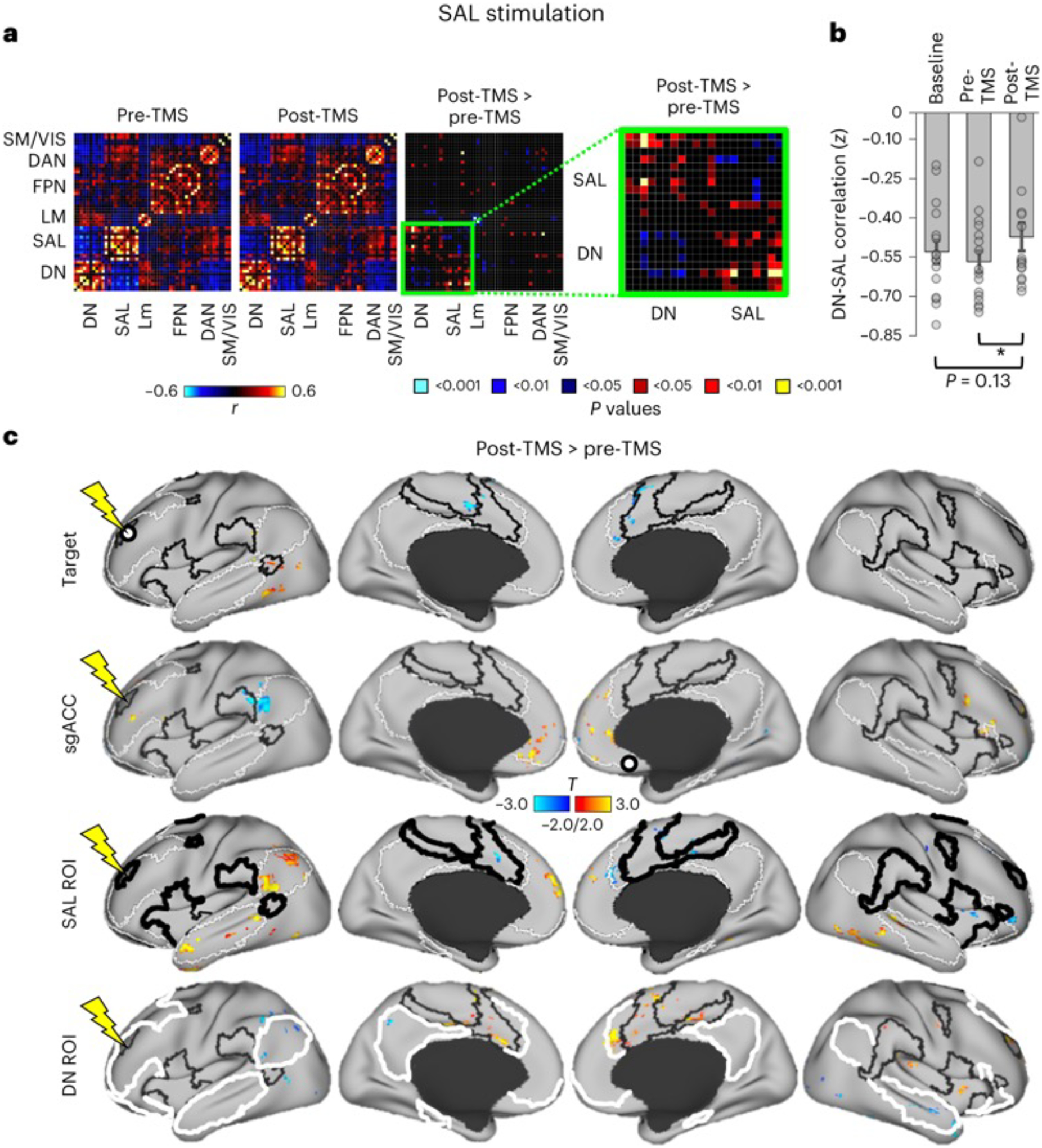
Stimulation of the SAL modestly increased FC between salience and DNs. **a**, Pair-wise correlation matrices showing FC between 51 ROIs (from Yeo et al.^32^) covering the entire brain, grouped into seven major networks. Matrices represent FC defined using data collected before (pre-TMS) and after stimulation (post-TMS; colours represent *r* values), and their direct comparison (post-TMS > pre-TMS; colours represent uncorrected *P* values from paired *t*-tests). The direct comparison matrix (highlighted by a zoomed in matrix in green) shows that correlations between regions of the DN and SAL— which were negatively correlated in both pre-TMS and post-TMS conditions— increased their FC following SAL stimulation. **b**, Bar graphs show the average correlation between the whole-brain network ROIs for the DN and SAL at baseline, pre-TMS and post-TMS. Grey dots represent FC changes within each subject (*n* = 16 in total). Statistical testing involved a paired two-sided *t*-test uncorrected for multiple comparisons: for post-TMS > baseline, *P* = 0.13; for post-TMS > pre-TMS, *P* = 0.03. *P < 0.05. Error bars represent ±standard error of the mean. Significant FC increases were noted between pre-TMS and post-TMS, but not between baseline and post-TMS. **c**, Surface projections of voxel-wise statistical *T* maps (*P* < 0.05, FWE cluster corrected) comparing FC from different seed regions (post-TMS > pre-TMS). Seed-based FC maps were defined using each individual’s SAL target region (target), using a seed relegated to the sgACC, or using the whole-brain network maps from Yeo et al.^32^ with the SALs (SAL ROI) and DNs (DN ROI) as seeds. Black outlines indicate SAL boundaries and white outlines indicate DN boundaries. Seeds are indicated by white circles with black outlines (targets and sgACC) or thick outlines (SAL ROI and DN ROI). Negligible FC changes were observed when the target or sgACC were used as seeds. When the full SAL was used as the seed, SAL target stimulation led to modest FC increases mostly located within the boundaries of the DN (see small yellow–red regions in the third row). Conversely, modest increases in the FC of the DN ROI were observed, located in regions mostly falling within the SAL (small yellow–red regions in the fourth row). LM, limbic network; SM/VIS, somatomotor and visual networks.

**Fig. 6 |:**
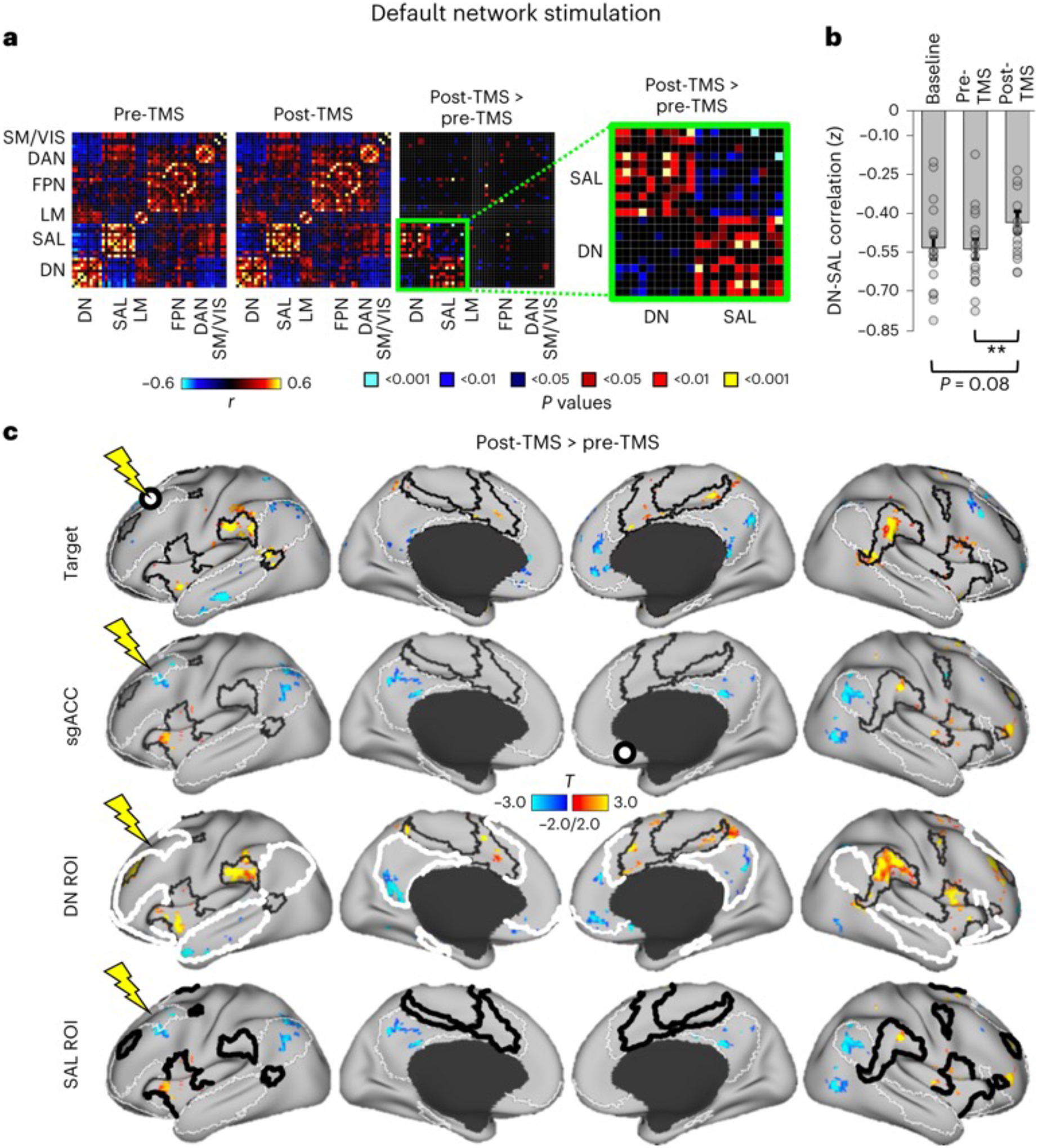
Stimulation of the DN markedly increased FC between DNs and SALs. **a**, Pair-wise correlation matrices (as in Fig. 5) following DN stimulation. The direct comparison matrix (highlighted by a zoomed in matrix in green) shows that correlations between regions of the DNs and SALs—which were negatively correlated in both pre-TMS and post-TMS conditions—became substantially less anti-correlated following DN stimulation. **b**, Bar graphs (as in Fig. 5) following DN stimulation. Grey dots represent FC changes within each subject (*n* = 16 in total). Statistical testing involved a paired two-sided *t*-test uncorrected for multiple comparisons: for post-TMS > baseline, *P* = 0.08; for post-TMS > pre-TMS, *P* = 0.009. **P < 0.01. Error bars re*p*resent ±standard error of the mean. Significant FC increases were noted between pre-TMS and post-TMS, but not between baseline and post-TMS (although this trended toward significance). **c**, Surface projections of voxel-wise statistical *T* maps (*P* < 0.05, FWE cluster corrected) comparing FC from different seed regions post-TMS > pre-TMS. Seed-based FC maps were defined using each individual’s DN target region (target), using a seed relegated to the sgACC, or using the whole-brain network maps from Yeo et al.^32^ for the DNs (DN ROI) and SALs (SAL ROI) as seeds. Again, white outlines indicate boundaries of the DN and black outlines indicate boundaries of the SAL. Seeds are indicated by white circles with black outlines (Targets and sgACC) or thick outlines (DN ROI and SAL ROI). FC increases were observed in the SAL when the target or sgACC were used as seeds. When the full DN was used as the seed, DN target stimulation led to FC increases that were mostly located within the boundaries of the SAL (see small yellow–red regions in third row) and modest FC decreases in the DN (see small blue regions in fourth row). Using the SAL ROI as a seed, dramatic FC increases were observed with the DN (yellow–red regions) and modest FC decreases (blue regions) were noted within the SAL. LM, limbic network; SM/VIS, somatomotor and visual networks.

We present evidence supporting that dlPFC TMS can lead to different effects depending on the network identity of the site of stimulation. Identical TMS patterns, applied within the same subjects (Fig. 1) to nearby prefrontal regions (Fig. 2), led to opposite effects on local (that is, near to the stimulation site) metabolism (Fig. 3). TMS targeted to the SAL increased local glucose metabolism while TMS targeted to the DN decreased local metabolism. Stimulation of both sites also led to broad distributed increases in metabolism that overlapped with regions of the SAL, but this effect was greater when stimulation was targeted to the SAL itself (Fig. 4). We further observed that stimulation of both SAL and DN targets affected the level of anti-correlation between the two networks. That is, TMS caused these networks, which are typically anti-correlated (Fig. 2 and Supplementary Fig. 1), to increase their FC with one another (that is, to become less anti-correlated). (Figs. 5 and 6). However, these effects were quantitatively different across conditions, as DN-TMS led to considerably more profound FC increases between the two networks (Fig. 6). Finally, we show evidence that, across subjects, the magnitude of stimulation-induced change in local glucose metabolism was correlated with the magnitude of changes in FC between regions of the SAL and DN networks, and between the sgACC and these networks (Supplementary Figs. 5 and 6). This suggests that TMS has more pronounced effects on distal brain regions when local activity and metabolism are more robustly perturbed. In other words, optimizing local effects of TMS may also optimize long-range effects. Taken together, these findings support that focal non-invasive brain stimulation can lead to changes in brain activity that propagate across distributed networks^6,7^—but extends this by showing that the effects can differ across networks. Finally, our results invoke putative mechanisms of action of TMS in treating depression. More specifically, TMS may act through decreasing functional couplings, including decreasing anti-correlation between distinct but anti-correlated networks—with specific regional FC changes dependent on the network stimulated. This hypothesis is supported by studies in MDD. For example, effective TMS courses in MDD are associated with FC reductions in the DN, in the SAL network^53^, and between the sgACC and DN^52^.

### Possible explanation of the observed local effects of TMS

The present results challenge the commonplace assumption that TMS at a given frequency induces uniform changes in cortical activity, independent of the region (or network) being stimulated^15,18^. Although 20 Hz TMS was expected to increase metabolism locally, we observed this only following TMS at SAL targets, with decreased local metabolism observed following TMS at DN targets (Fig. 3). This double-dissociation provides stronger evidence for opposing effects than if a null result had been observed following DN stimulation.

There are several reasons why we may have observed differential local effects of 20 Hz TMS in the DNs and SALs. Generally, local reactivity to TMS can very across individuals^54^. This may be due to genetic differences, differences in the baseline FC or differences in the neuronal subpopulations being stimulated across individuals^15,55^. Gross anatomical variables can also determine local responsiveness to TMS, such as scalp-to-cortical distance and gyral morphology^15^. Here, we did not observe systematic differences in these features when we visually assessed cortical anatomy at the target sites (Supplementary Fig. 3). However, the two stimulation sites differed markedly in functional organization, as they were selected to be in distinct, anti-correlated large-scale networks. There is evidence from intra-cranial recordings that different large-scale networks may operate preferentially at specific frequencies^35^. One hypothesis is that 20 Hz stimulation may affect the DNs and SALs in different ways due to these networks’ different intrinsic oscillatory properties. In addition, cortical reactivity to TMS depends on the connectivity and cytoarchitecture of the stimulated region^15^. Therefore, local changes in cortical activity could be subject to idiosyncratic differences in connectivity and cytoarchitecture between default and salience regions. Other potential factors are described below in ‘Limitations and potential confounds’.

### Possible explanation of the distributed FC effects of TMS

Similar to local changes, the directionality of distributed effects resulting from TMS may depend on several factors, for example, the long-distance structural and FC of the stimulation site, state dependency and/or the pattern of TMS employed. Although purely speculative, our FC findings may also be related to endogenous neural oscillations. There is evidence that the low-frequency BOLD signals that contribute to FC may be linked to neural oscillations^36,39,56–58^. Given evidence that different networks may operate at different frequencies^37,38^, the DNs and SALs may differ in their response to 20 Hz stimulation on the basis of their intrinsic oscillatory properties. This remains to be tested in a future study that can record high-frequency activity and how it is affected by TMS. Indeed, other studies have suggested that synchronization or de-synchronization of endogenous neural oscillations could be a mechanism of the anti-depressant effects of dlPFC TMS^59–61^.

The inter-network FC effects we observed may be explained by the existence of bi-directional functional influences between the DNs and SALs. There is evidence that intra-cortical stimulation of the DNs and SALs in humans leads to effects that propagate between networks^62^. Further, the SAL has repeatedly been shown to mediate interactions between other networks^63^. Moreover, consistent with our results, Tik et al. found that high-frequency TMS to left dlPFC selectively changed FC between anterior portions of the DNs and SALs^48^.

### Putative clinical and behavioural significance

While we did not directly test the behavioural and therapeutic consequences of TMS, our findings may have important ramifications for clinical and behavioural paradigms. First, there are implications for targeting in clinical TMS paradigms. Currently, the dlPFC target for TMS is established through crude anatomical landmarks, for example, at a site 5 cm anterior to the location where the motor threshold is established. This results in extensive variability in the network that is targeted in dlPFC across individuals. Our results support the notion that the effects of TMS can vary on the basis of the network that is targeted. If validated by further studies—particularly those in clinical cohorts—this may explain why certain depressed individuals respond less to TMS, that is, because in those individuals the network that is most critical to the therapeutic effects of TMS is not being stimulated. This would represent a paradigm shift in TMS clinical practice in which TMS clinicians maximally target a specific network in a given individual.

Second, extant studies have demonstrated abnormal FC in MDD. Relevant to our findings, these abnormalities consistently include increased FC within the DN^52,64–67^, increased FC within the SAL^53,64^, increased FC between the sgACC and DN^52^, and decreased FC (that is, heightened anti-correlations) between the DNs and SALs^68–70^. Furthermore, efficacious courses of TMS in depressed patients reverse some of these FC abnormalities, as well as others^45,52,53,71^. Consistent with this being a putative mechanism of its anti-depressant effects, we found that TMS decreased FC within the DNs and SALs (following DN-TMS) and decreased FC between the networks (in both conditions, but more so following DN-TMS). Also, in both conditions, TMS reduced FC between the sgACC and the DNs and SALs in a manner that was dependent on the magnitude of local metabolic effects. While our FC findings are consistent with what has been observed in clinical cohorts, conclusions drawn from this work must be qualified by the fact that we studied healthy individuals. That is, FC abnormalities in depressed subjects may have altered their functional responses to our specific TMS protocol.

### Implications for studying networks across spatial levels

We observed causal relationships between local changes in metabolism and selected FC changes. Other studies have described correlations between local FDG-PET metabolism and FC^72–74^. For example, local glucose consumption in the DN is correlated to FC within this network^72,73^. Notably, our study is among the first to demonstrate causality between local metabolism changes and FC changes by using an exogenous perturbation such as TMS. This supports the idea that local changes in neural activity can lead to network-wide effects, bridging spatial scales, and that local cortical activity places functional constraints on distributed FC^74,75^. For instance, the degree of local synaptic activity at a given region correlates to the amount of long-range inputs this region receives^74^, and local neurotransmitter receptor density can influence FC^76^. Our findings strengthen the hypothesis that local activity plays a determinative role in shaping distributed function. This has practical ramifications for experimental and clinical TMS paradigms: It suggests that by more specifically and more robustly modulating cortical targets, more profound FC changes can be achieved.

### Limitations and potential confounds

A notable limitation of the current study is the absence of a sham condition. We chose not to include a sham condition, and to instead compare two active controls, because an added condition would expose subjects to additional radiation. As a result, our findings (for example, increased SAL metabolism in both conditions, Fig. 4) may be partially explained by non-specific effects of receiving TMS, such as somatosensory, pain or novelty effects—all of which are relevant to the proposed functions of the SAL^77^. Indeed, some subjects informally reported finding the SAL-TMS condition uncomfortable (and then stated that this discomfort ceased immediately after stimulation). However, it is unlikely that such non-specific effects fully account for our findings. First, prominent portions of the pain matrix did not increase their metabolism following TMS, for example, the posterior insula, thalamus and primary sensory cortex^78^. Second, FC changes (Figs. 5 and 6) were measured when subjects were no longer receiving TMS and during which they experienced no discomfort. Third, session order was counter-balanced across subjects, arguing against novelty as a factor. Fourth, metabolic changes at the specific targets correlated with the observed FC changes, suggesting that observed changes were driven by neural stimulation *at the* target specifically, as opposed to being driven by non-specific global effects. Additionally, there is the issue of whether the inclusion of a sham condition is a suitable control, as sham stimulation may not produce the same somatosensory impact as real TMS^79^.

A second potential limitation is that due to the long half-life of ^18^F radiotracers, we could not perform FDG-PET imaging immediately before and after TMS, as we did with fMRI. This necessitated that FDG-PET comparisons were made across several days (that is, post-TMS > baseline), while FC comparisons occurred on the same day. However, this concern is mitigated by the fact that regional estimates of glucose metabolism are normally remarkably stable across time within individuals^80,81^ (see also FDG-PET repeatability measures in Methods) and by the fact that we observed some consistency in the effects of TMS across different baselines (Supplementary Fig. 4). Third, our small sample size (16 subjects carried to the main analysis) limits the generalizability of our findings. Fourth, because dlPFC contains closely juxtaposed regions of discrete networks^31^, and because the E-field induced by TMS with the coils we used are not completely focal^82^, the induced E-field at the non-stimulated target was not zero (Supplementary Fig. 8). As such, there is the possibility of incomplete stimulation independence at each target from the other target. Fifth, while our results have clinical implications, elements of our design limit its applicability for clinical TMS paradigms. Specifically, we used different stimulation parameters than those traditionally used clinically (20 Hz as opposed to 10 Hz (ref. 4) or intermittent theta burst stimulation^83^). We also studied the effects of only one TMS session, while clinical paradigms typically employ several sessions that may induce more neuroplastic local and distributed changes than we were able to capture. Finally, we studied a healthy cohort, as opposed to MDD patients whose functional pathology could have affected our results.

### Future directions

Future studies are needed to confirm the differential local metabolic effects of TMS we observed. While we compared local and distributed effects in different networks with the same TMS pattern, future studies could examine how different TMS patterns to the same network alter local and distributed activity. Intra-cranial stimulation and recordings could be used to probe whether TMS disrupts FC by de-synchronizing neuronal oscillations, as we speculate. Finally, the advent of high-resolution precision network mapping in the individual^12,31,50^ could be used to strengthen TMS targeting approaches, leading to more reliable and more selective network engagement. These endeavours could lead to more detailed network mechanisms of actions of TMS that could be exploited to decrease or increase functional couplings depending on the investigational question or clinical condition to be treated.

## Methods

### Subjects

Twenty healthy subjects (mean age 23.85 years, s.d. 3.8 years, 13 female) participated in the study. All subjects were screened by author M.C.E. (board certified in both neurology and psychiatry) to ensure that they had no neurological or psychiatric history, no symptoms of active depression (as assessed by the 17-item Hamilton Depression Rating Scale, mean score of 0.1) and were not taking any psychotropic medications. One subject was excluded because PET data acquisition failed due to a software malfunction during one of their post-TMS FDG-PET sessions. Two additional subjects were excluded on the basis that one of their derived targets was anatomically distant from the rest of the group. Specifically, one subject’s SAL target, and another person’s DN target, were each located in the frontal eye fields, when the same procedures used in other subjects were followed. A fourth subject was excluded due to excessive head motion during imaging (see quality control procedures described below). Therefore, 16 subjects were carried through to the main analyses. Subjects provided written informed consent and the study was approved by the Mass General Brigham Human Research Committee (the Institutional Review Board of the Massachusetts General Hospital).

### Sessions

The overall experimental design is depicted in Fig. 1. All experimental procedures were conducted at the Athinoula A. Martinos Center for Biomedical Imaging/Massachusetts General Hospital in the MR-PET suite. Subjects participated in three experimental visits on separate days: a ‘baseline’ visit and two ‘experimental’ visits. During the baseline visit, subjects underwent a combined FDG-PET–fMRI imaging session. This baseline data were used to functionally determine, on an individual-subject basis, the locations of the DN and SAL targets (see ‘Definition of TMS targets’ below). The two separate experimental visits occurred on separate days at least 6 days apart (mean 32 days, median 23 days, range 6–236 days). During each experimental visit, subjects first underwent an fMRI imaging session, then received 20 Hz repetitive TMS at one of the pre-defined target sites (SAL or DN), then immediately underwent a combined FDG-PET–fMRI imaging session. The order of stimulated targets (SAL versus DN) was counter-balanced across subjects, such that half of subjects received SAL stimulation in their first experimental visit, while the other half received DN stimulation first. This paradigm recapitulates our previous experimental designs^27,28^. Notably, when 4 of the initial 20 subjects were removed, there was a slight counter-imbalance, with 9 of the remaining subjects receiving DN-TMS first and 7 of the remaining subjects receiving SAL-TMS first.

### MRI acquisition

MRI data were acquired using a 3.0 T whole-body scanner (Siemens), equipped for echo-planar imaging with a 12-channel 3-axis gradient head coil. Head movements were restricted using foam cushions. For each fMRI scanning session, one structural scan (~8 min) and three consecutive fMRI BOLD resting-state runs (6 min each, 18 min per fMRI session) were performed. In the experimental visits, to minimize the time between TMS and FDG-PET–fMRI and to capture the effects of neuromodulation, the three BOLD runs were acquired first, immediately after the localizer. Mean time from the completion of TMS to the start of FDG-PET–fMRI was 370 s (s.d. ±78 s) for the SAL-TMS condition and 326 s (s.d. ±47 s) for the DN-TMS condition. This is within the expected duration of effects of TMS, which can last up to 1 h (ref. 84). Structural images were acquired via a T1-weighted 3D magnetization prepared rapid gradient echo (MPRAGE) image, acquired with the following parameters: echo time (TE) 1.64 ms, repetition time (TR) 2,530 ms, TI 1,200 ms, flip angle 7°, voxel size 1 × 1 × 1 mm, FOV 280 × 280, and 208 frames. BOLD data were acquired using the following parameters: TR 3,000 ms, TE 30 ms, flip angle 90°, voxel size 3.375 × 3.375 × 3.0 mm, field-of-view (FOV) 448 × 448, 120 frames and no acceleration factor. A fixation dot (a small white dot centred on a black background) was presented to subjects on a screen via a rear projection system. Participants were instructed to stay awake, remain extremely still and to stare at the fixation dot during imaging.

### FDG-PET acquisition

FDG-PET images were acquired during fMRI scanning on a BrainPET prototype (Siemens Healthineers). FDG-PET procedures were identical across the subjects’ three FDG-PET–fMRI scans (baseline and two experimental sessions). Following their pre-TMS fMRI scan, subjects were injected with ^18^Fluorodeoxyglucose: average 188 MBq (range 164–212 MBq), average 5.1 mCi (range 4.4–5.7 mCi). TMS was started approximately 7 min after injection (mean ± s.d.: 462 ± 252 s for SAL-TMS and 390 ± 102 s for DN-TMS) to allow time for the radioligand to circulate and best capture the effects of TMS on FDG uptake. Approximately 34 min after injection (mean in baseline visit 33.0 min, SAL-TMS visit 34.3 min and DN-TMS visit 34.3 min), PET data were collected for a duration of approximately 36 min (up to a timepoint of 70 min post-injection). Data were stored in listmode format.

### Definition of TMS targets

Baseline fMRI data were used to define, on the basis of FC estimates, two dlPFC targets in each individual. To identify the two targets, we used a procedure developed by Fox and colleagues^40^. Specifically, we used a group-averaged seed-based FC map of the sgACC (sgACC FC map) as a seed for FC in all subjects. This map was derived by Fox et al.^40^ by first defining a 10 mm spherical ROI in the right sgACC (MNI coordinates: 6, 16, −10), with the location of this based on prior studies. This ROI was then used by Fox et al.^40^ as a seed to generate a seed-based FC map in a cohort of 98 individuals (a subset of the 1,000 individuals in Yeo et al.^32^). So as not to bias dlPFC target selection, a large dlPFC ROI was removed from this map. Of note, this ROI encompassed medial portions of prefrontal cortex that are often designated as part of dorsomedial prefrontal cortex. The resultant map was then itself used as a seed to generate FC maps in the 16 subjects of this study. Again, repeating procedures in Fox et al.^40^, we calculated FC strength between this sgACC FC map and 163 nodes, 4 mm in diameter, covering the left dlPFC. The centre of the node with the most positive and the most negative FC value with the sgACC FC map was chosen as the DN target and SAL target, respectively, for that individual. The DN target was consistently located in the dorsomedial prefrontal portion of the DN (BA 9, medial superior frontal gyrus), while the SAL target was typically situated in the lateral prefrontal node of the SAL (BA 9/46 or BA 46, lateral middle frontal gyrus) with some targets falling into the surrounding FPN region in the lateral frontal cortex (Fig. 2). The mean Euclidean distance between the two targets across subjects was 33 mm.

Our rationale for using this targeting paradigm was threefold. First, an sgACC FC map was chosen because multiple studies support the clinical importance of the sgACC in MDD^85,86^. Second, we used a sgACC FC map, as opposed to direct correlations between target locations and the sgACC ROI, because negative correlations from this region could be compromised by susceptibility issues, markedly driving down signal to noise^40^. Third, the sgACC FC map allowed us to delineate regions showing both positive and negative correlations, which fell within the demarcations of two large-scale, anti-correlated networks (Fig. 2). This further allowed us to study the effects of stimulating two adjacent and anti-correlated distributed networks and assess the effects of targeted stimulation in a network-informed manner.

### TMS administration

Repetitive TMS was applied using a MagPro X100 Stimulator with a MagPro Cool B-65 coil or a MagPro MCF-65 coil, depending on coil availability. Notably, these two coils have identical windings and geometry and thus deliver identical stimulation effects. The coils differ only with respect to cooling mechanism (B65: active; MCF-65: static), and small differences in maximal initial dB/dt (B65: 36 kT s^−1^; MCF-65: 32 kT s^−1^) and biphasic pulse width (B65: 290 μs; MCF-65: 280 μs). Importantly, during TMS, accuracy and reproducibility of coil location and orientation was ensured with a frameless stereotactic neuronavigation system (Nexstim NBS). Immediately before each TMS application, the resting motor threshold (RMT) was obtained by administering single pulses (with the same coil used for stimulation) delivered to the hand knob in the left primary motor cortex (determined through neuronavigation). The RMT was defined as the minimum total machine output required to elicit a motor evoked potential ≥50 μV in the contralateral (right) first dorsal interosseous muscle, 50% of the time. Mean RMT across subjects was 52% of the total machine output for both target sessions and did not differ across the two target sessions (*P* = 0.70). For both stimulation sites, TMS was applied as high-frequency (20 Hz) stimulation at 110% of RMT, 40 pulses per train, with an inter-train interval of 28 s for 45 total trains (1,800 total pulses, 22.5 min). These parameters are within recommended safety limits for TMS^17^ and were the exact parameters used in our prior protocol^27^. Moreover, 20 Hz TMS has been used to successfully treat depressive symptoms^87^. Mean (± s.d.) time from the completion of TMS to the start of FDG-PET/fMRI imaging was 326 ± 47 s for the DN-TMS condition and 370 ± 78 s for the SAL-TMS condition.

### Computational estimation of intra-cranial electrical fields

E-fields distributions for 15 out of 16 subjects are shown in Supplementary Fig. 8. To estimate the intra-cranial E-field distributions, we utilized our recently developed Boundary Element Modelling (BEM) approach accelerated by the Fast Multpole Method (FMM)^88,89^. The publicly available MATLAB-based BEM-FMM toolbox was used to perform the E-field estimation as described in full detail in Makarov et al.^88^. The BEM-FMM method requires surface meshes of the tissue conductivity boundaries (skin, skull, cerebrospinal fluid, grey matter and white matters) to be extracted from the individual subjects’ MRI data. As a pre-processing step, we applied bias correction to the T1-weighted MRI data using SPM12 to facilitate tissue segmentation. Subsequently, we employed SIMNIBS^90,91^ (version 3.1) and ran both ‘mri2mesh’ and ‘headreco’ pipelines and visually inspected the resulting surface mesh quality. For each subject, the meshes with the best quality from each pipeline were selected for the final computations. The position and orientation of the TMS coil for each target were exported from the neuronavigation system (Nexstim NBS) and subsequently transformed to the coordinates of the surface meshes obtained from SIMNIBS. Finally, the BEM-FMM solver was executed for each target location and the E-field distributions were evaluated on the grey–white matter boundary surface and visualized in MATLAB.

### ROI selection

We assessed the topography of metabolic and FC changes at the network level using whole-network ROIs at seven major networks (salience, default, frontoparietal control, dorsal attention, somatomotor, limbic and visual) defined by the parcellation by Yeo et al.^32^. We also used contiguous smaller ROIs of these networks defined by Yeo et al.^32^ (*n* = 51) for region-level analyses, as well as a 10 mm sgACC seed defined by Fox et al.^40^. We particularly focused our region-level analysis on the 11 ROIs making up the SAL and the 10 ROIs making up the DN. Notably, while our analysis used the Yeo et al. parcellation, several other parcellation schemes have comparable representations of the DNs and SALs, including the representations of these systems in dlPFC (Supplementary Fig. 9).

### FC analysis

BOLD data were first pre-processed using spatial normalization directly to a standard MNI 152 template brain, as well as motion and slice timing corrected using a combination of software packages: Statistical Parametric Mapping software package (Wellcome Department of Cognitive Neurology), FSL (the FMRIB Software Library), MATLAB (Mathworks) and FreeSurfer (http://surfer.nmr.mgh.harvard.edu). Registration was performed with linear regression with the SPM software package. The following nuisance variables and their temporal derivatives were regressed during pre-processing: mean whole-brain signal, six motion parameters, mean white matter signal and mean ventricular signal as described in ref. 32. Additionally, data were low-pass filtered to exclude signals above 0.08 Hz. Smoothing was performed with a 6 mm full-width-at-half-maximum Gaussian blur. Following pre-processing, volumetric seed-based FC analyses were conducted by extracting the BOLD time course from a seed or ROI (for example, those defined in Yeo et al.^32^) and calculating the *z*-transformed Pearson’s product moment correlation coefficient between this seed/ROI and all other brain voxels, as described in ref. 92. Correlations between pairs of ROIs were calculated by correlating the average signal within each of the ROIs. FC maps were projected to the surface for visualization using the Connectome Workbench (version 1.4) toolbox (www.humanconnectome.org/software/connectome-workbench).

### FDG-PET analysis

PET images were reconstructed using an ordered-subsets expectation maximization algorithm using 6 iterations and 16 subsets, and correcting for random coincidences, dead time, isotope decay, detector sensitivity, photon attenuation and scatter. Attenuation correction was provided via a validated and highly reproducible SPM-based method^93,94^ (see also FDG-PET repeatability measures section). Static images were reconstructed 45–65 min post-injection to best capture the glucose metabolism changes induced by TMS stimulation. The reconstructed PET volume consisted of a 256 × 256 × 153 matrix of 1.25 mm isotropic voxels. To avoid potential subject head motion biasing the PET image quantification, motion correction was enabled into the PET reconstruction using a dual-pass image reconstruction method. Finally, the PET images were co-registered back into the MPRAGE images to allow ideal alignment of both image techniques. This was the same MPRAGE used to align BOLD data. Images were spatially normalized into the Montreal Neurological Institute (MNI) space using the Dartel toolbox in SPM. This spatial normalization enabled analysis using fMRI-derived and pre-defined atlas ROIs. Finally, ROI-based PET values were intensity normalized using the whole-brain as the reference region.

### Statistical testing

Changes in target metabolism (WBn-suv values) before and after TMS were compared with paired *t*-tests. Paired *t*-tests were also used to compare FC changes (*z* values) before and after TMS between ROIs. Effect sizes of TMS induced changes in metabolism and FC were computed as Cohen’s *d* values. FC maps were thresholded at a *P* value of <0.05, cluster corrected for family-wise error (FWE). Correlations between TMS-induced changes in WBn-suv values and *z* values were calculated with Pearson correlations (*r* and *R*^2^).

### Quality control of BOLD data

As head motion has a profound effect on FC^95^, we employed stringent quality control of the BOLD data. Each run was evaluated for slice-based temporal signal-to-noise ratio (SNR), mean and maximum relative motion, mean and maximum absolute motion, and relative motion greater than 0.1 mm and 0.5 mm. A given run was excluded from the analysis if the temporal SNR for that run was lower than 2 s.d. below the group mean of all runs, or if there were more than five movements greater than 0.5 mm in that run. By these criteria, only one subject was affected, with three of their nine BOLD runs being unusable, leading to that subject’s exclusion from further analysis on the basis of head motion.

### FDG-PET repeatability measures

Intra-scanner reproducibility for a subset of this PET dataset (13 out of 20 subjects) has been published elsewhere^93^. Briefly, we assessed relative changes, intra-class correlation coefficient, reproducibility coefficient and Bland–Altman limits of agreement to assess repeatability across scans. This revealed minimal, insignificant relative changes across the three PET acquisitions (*P* = 0.90).

## Supplementary Material

Supplementary Material

## Data Availability

Study data available at https://openneuro.org/datasets/ds004552/versions/1.0.0.

## References

[1] Perera T The Clinical TMS Society consensus review and treatment recommendations for TMS therapy for major depressive disorder. Brain Stimul. 9, 336–346 (2016). 10.1016/j.brs.2016.03.010 PMC5612370 27090022

[2] Voigt JD, Leuchter AF & Carpenter LL Theta burst stimulation for the acute treatment of major depressive disorder: a systematic review and meta-analysis. Transl. Psychiatry 11, 330 (2021). 10.1038/s41398-021-01441-4 PMC8163818 34050123

[3] Eche J Low- vs high-frequency repetitive transcranial magnetic stimulation as an add-on treatment for refractory depression. Front. Psychiatry 3, 13 (2012). 10.3389/fpsyt.2012.00013 PMC3296064 22408627

[4] O’Reardon JP Efficacy and safety of transcranial magnetic stimulation in the acute treatment of major depression: a multisite randomized controlled trial. Biol. Psychiatry 62, 1208–1216 (2007). 10.1016/j.biopsych.2007.01.018 17573044

[5] Cole EJ Stanford neuromodulation therapy (SNT): a double-blind randomized controlled trial. Am. J. Psychiatry 179, 132–141 (2022). 10.1176/appi.ajp.2021.20101429 34711062

[6] Fox MD, Halko MA, Eldaief MC & Pascual-Leone A Measuring and manipulating brain connectivity with resting state functional connectivity magnetic resonance imaging (fcMRI) and transcranial magnetic stimulation (TMS). NeuroImage 62, 2232–2243 (2012). 10.1016/j.neuroimage.2012.03.035 PMC3518426 22465297

[7] Momi D Perturbation of resting-state network nodes preferentially propagates to structurally rather than functionally connected regions. Sci. Rep 11, 12458 (2021). 10.1038/s41598-021-90663-z PMC8203778 34127688

[8] Salomons TV Resting-state cortico-thalamic-striatal connectivity predicts response to dorsomedial prefrontal rTMS in major depressive disorder. Neuropsychopharmacology 39, 488–498 (2014). 10.1038/npp.2013.222 PMC3870791 24150516

[9] Baeken C, Duprat R, Wu GR, De Raedt R & van Heeringen K Subgenual anterior cingulate-medial orbitofrontal functional connectivity in medication-resistant major depression: a neurobiological marker for accelerated intermittent theta burst stimulation treatment? Biol. Psychiatry Cogn. Neurosci. Neuroimaging 2, 556–565 (2017). 10.1016/j.bpsc.2017.01.001 29560909

[10] Baeken C Accelerated HF-rTMS in treatment-resistant unipolar depression: insights from subgenual anterior cingulate functional connectivity. World J. Biol. Psychiatry 15, 286–297 (2014). 10.3109/15622975.2013.872295 24447053

[11] Weigand A Prospective validation that subgenual connectivity predicts antidepressant efficacy of transcranial magnetic stimulation sites. Biol. Psychiatry 84, 28–37 (2018). 10.1016/j.biopsych.2017.10.028 PMC6091227 29274805

[12] Cash RFH Using brain imaging to improve spatial targeting of transcranial magnetic stimulation for depression. Biol. Psychiatry 90, 689–700 (2021). 10.1016/j.biopsych.2020.05.033 32800379

[13] Fox MD, Buckner RL, White MP, Greicius MD & Pascual-Leone A Efficacy of transcranial magnetic stimulation targets for depression is related to intrinsic functional connectivity with the subgenual cingulate. Biol. Psychiatry 72, 595–603 (2012). 10.1016/j.biopsych.2012.04.028 PMC4120275 22658708

[14] Miron JP, Jodoin VD, Lesperance P & Blumberger DM Repetitive transcranial magnetic stimulation for major depressive disorder: basic principles and future directions. Ther. Adv. Psychopharmacol 11, 20451253211042696 (2021). 10.1177/20451253211042696 PMC8474312 34589203

[15] Valero-Cabre A, Amengual JL, Stengel C, Pascual-Leone A & Coubard OA Transcranial magnetic stimulation in basic and clinical neuroscience: a comprehensive review of fundamental principles and novel insights. Neurosci. Biobehav. Rev 83, 381–404 (2017). 10.1016/j.neubiorev.2017.10.006 29032089

[16] Huang YZ, Edwards MJ, Rounis E, Bhatia KP & Rothwell JC Theta burst stimulation of the human motor cortex. Neuron 45, 201–206 (2005). 10.1016/j.neuron.2004.12.033 15664172

[17] Rossi S, Hallett M, Rossini PM & Pascual-Leone A, & Safety of TMS Consensus Group. Safety, ethical considerations, and application guidelines for the use of transcranial magnetic stimulation in clinical practice and research. Clin. Neurophysiol 120, 2008–2039 (2009). 10.1016/j.clinph.2009.08.016 PMC3260536 19833552

[18] Valero-Cabre A, Payne BR & Pascual-Leone A Opposite impact on ^14^C-2-deoxyglucose brain metabolism following patterns of high and low frequency repetitive transcranial magnetic stimulation in the posterior parietal cortex. Exp. Brain Res 176, 603–615 (2007). 10.1007/s00221-006-0639-8 16972076

[19] Kito S, Hasegawa T & Koga Y Neuroanatomical correlates of therapeutic efficacy of low-frequency right prefrontal transcranial magnetic stimulation in treatment-resistant depression. Psychiatry Clin. Neurosci 65, 175–182 (2011). 10.1111/j.1440-1819.2010.02183.x 21414091

[20] Kimbrell TA Left prefrontal-repetitive transcranial magnetic stimulation (rTMS) and regional cerebral glucose metabolism in normal volunteers. Psychiatry Res 115, 101–113 (2002). 10.1016/s0925-4927(02)00041-0 12208488

[21] Speer AM Opposite effects of high and low frequency rTMS on regional brain activity in depressed patients. Biol. Psychiatry 48, 1133–1141 (2000). 10.1016/s0006-3223(00)01065-9 11137053

[22] Eisenegger C, Treyer V, Fehr E & Knoch D Time-course of “off-line” prefrontal rTMS effects—a PET study. NeuroImage 42, 379–384 (2008). 10.1016/j.neuroimage.2008.04.172 18511301

[23] Knoch D Lateralized and frequency-dependent effects of prefrontal rTMS on regional cerebral blood flow. NeuroImage 31, 641–648 (2006). 10.1016/j.neuroimage.2005.12.025 16497518

[24] Ruff CC Concurrent TMS-fMRI and psychophysics reveal frontal influences on human retinotopic visual cortex. Curr. Biol 16, 1479–1488 (2006). 10.1016/j.cub.2006.06.057 16890523

[25] Ruff CC, Driver J & Bestmann S Combining TMS and fMRI: from ‘virtual lesions’ to functional-network accounts of cognition. Cortex 45, 1043–1049 (2009). 10.1016/j.cortex.2008.10.012 PMC2726131 19166996

[26] Bohning DE BOLD-f MRI response to single-pulse transcranial magnetic stimulation (TMS). J. Magn. Reson. Imaging 11, 569–574 (2000). 10.1002/1522-2586(200006)11:6<569::aid-jmri1>3.0.co;2-3 10862054

[27] Eldaief MC, Halko MA, Buckner RL & Pascual-Leone A Transcranial magnetic stimulation modulates the brain’s intrinsic activity in a frequency-dependent manner. Proc. Natl Acad. Sci. USA 108, 21229–21234 (2011). 10.1073/pnas.1113103109 PMC3248528 22160708

[28] Halko MA, Farzan F, Eldaief MC, Schmahmann JD & Pascual-Leone A Intermittent theta-burst stimulation of the lateral cerebellum increases functional connectivity of the default network. J. Neurosci 34, 12049–12056 (2014). 10.1523/JNEUROSCI.1776-14.2014 PMC4152606 25186750

[29] Li X Acute left prefrontal transcranial magnetic stimulation in depressed patients is associated with immediately increased activity in prefrontal cortical as well as subcortical regions. Biol. Psychiatry 55, 882–890 (2004). 10.1016/j.biopsych.2004.01.017 15110731

[30] Gratton C, Lee TG, Nomura EM & D’Esposito M The effect of theta-burst TMS on cognitive control networks measured with resting state fMRI. Front. Syst. Neurosci 7, 124 (2013). 10.3389/fnsys.2013.00124 PMC3874542 24416003

[31] Braga RM & Buckner RL Parallel interdigitated distributed networks within the individual estimated by intrinsic functional connectivity. Neuron 95, 457–471 (2017). 10.1016/j.neuron.2017.06.038 PMC5519493 28728026

[32] Yeo BT The organization of the human cerebral cortex estimated by intrinsic functional connectivity. J. Neurophysiol 106, 1125–1165 (2011). 10.1152/jn.00338.2011 PMC3174820 21653723

[33] Power JD Functional network organization of the human brain. Neuron 72, 665–678 (2011). 10.1016/j.neuron.2011.09.006 PMC3222858 22099467

[34] Doucet G Brain activity at rest: a multiscale hierarchical functional organization. J. Neurophysiol 105, 2753–2763 (2011). 10.1152/jn.00895.2010 21430278

[35] Hacker CD, Snyder AZ, Pahwa M, Corbetta M & Leuthardt EC Frequency-specific electrophysiologic correlates of resting state fMRI networks. NeuroImage 149, 446–457 (2017). 10.1016/j.neuroimage.2017.01.054 PMC5745814 28159686

[36] Foster BL, Rangarajan V, Shirer WR & Parvizi J Intrinsic and task-dependent coupling of neuronal population activity in human parietal cortex. Neuron 86, 578–590 (2015). 10.1016/j.neuron.2015.03.018 PMC4409557 25863718

[37] Kucyi A Intracranial electrophysiology reveals reproducible intrinsic functional connectivity within human brain networks. J. Neurosci 38, 4230–4242 (2018). 10.1523/JNEUROSCI.0217-18.2018 PMC5963853 29626167

[38] Kucyi A Electrophysiological dynamics of antagonistic brain networks reflect attentional fluctuations. Nat. Commun 11, 325 (2020). 10.1038/s41467-019-14166-2 PMC6965628 31949140

[39] Keller CJ Neurophysiological investigation of spontaneous correlated and anticorrelated fluctuations of the BOLD signal. J. Neurosci 33, 6333–6342 (2013). 10.1523/JNEUROSCI.4837-12.2013 PMC3652257 23575832

[40] Fox MD, Liu H & Pascual-Leone A Identification of reproducible individualized targets for treatment of depression with TMS based on intrinsic connectivity. NeuroImage 66, 151–160 (2013). 10.1016/j.neuroimage.2012.10.082 PMC3594474 23142067

[41] Hadas I Association of repetitive transcranial magnetic stimulation treatment with subgenual cingulate hyperactivity in patients with major depressive disorder: a secondary analysis of a randomized clinical trial. JAMA Netw. Open 2, e195578 (2019). 10.1001/jamanetworkopen.2019.5578 PMC6551850 31167023

[42] Fox MD The human brain is intrinsically organized into dynamic, anticorrelated functional networks. Proc. Natl Acad. Sci. USA 102, 9673–9678 (2005). 10.1073/pnas.0504136102 PMC1157105 15976020

[43] Walsh V & Cowey A Transcranial magnetic stimulation and cognitive neuroscience. Nat. Rev. Neurosci 1, 73–79 (2000). 10.1038/35036239 11252771

[44] Watanabe T Bidirectional effects on interhemispheric resting-state functional connectivity induced by excitatory and inhibitory repetitive transcranial magnetic stimulation. Hum. Brain Mapp 35, 1896–1905 (2014). 10.1002/hbm.22300 PMC6869044 23897535

[45] Philip NS Network mechanisms of clinical response to transcranial magnetic stimulation in posttraumatic stress disorder and major depressive disorder. Biol. Psychiatry 83, 263–272 (2018). 10.1016/j.biopsych.2017.07.021 PMC6679924 28886760

[46] Kang JI Frontostriatal connectivity changes in major depressive disorder after repetitive transcranial magnetic stimulation: a randomized sham-controlled study. J. Clin. Psychiatry 77, e1137–e1143 (2016). 10.4088/JCP.15m10110 27379563

[47] Beynel L, Powers JP & Appelbaum LG Effects of repetitive transcranial magnetic stimulation on resting-state connectivity: a systematic review. NeuroImage 211, 116596 (2020). 10.1016/j.neuroimage.2020.116596 PMC7571509 32014552

[48] Tik M Towards understanding rTMS mechanism of action: stimulation of the DLPFC causes network-specific increase in functional connectivity. NeuroImage 162, 289–296 (2017). 10.1016/j.neuroimage.2017.09.022 28912081

[49] Shang Y Theta-burst transcranial magnetic stimulation induced functional connectivity changes between dorsolateral prefrontal cortex and default-mode-network. Brain Imaging Behav 14, 1955–1963 (2020). 10.1007/s11682-019-00139-y 31197581

[50] Gordon EM Precision functional mapping of individual human brains. Neuron 95, 791–807 (2017). 10.1016/j.neuron.2017.07.011 PMC5576360 28757305

[51] Glasser MF A multi-modal parcellation of human cerebral cortex. Nature 536, 171–178 (2016). 10.1038/nature18933 PMC4990127 27437579

[52] Liston C Default mode network mechanisms of transcranial magnetic stimulation in depression. Biol. Psychiatry 76, 517–526 (2014). 10.1016/j.biopsych.2014.01.023 PMC4209727 24629537

[53] Godfrey KEM, Muthukumaraswamy SD, Stinear CM & Hoeh N Decreased salience network fMRI functional connectivity following a course of rTMS for treatment-resistant depression. J. Affect. Disord 300, 235–242 (2022). 10.1016/j.jad.2021.12.129 34986371

[54] Hinder MR Inter- and intra-individual variability following intermittent theta burst stimulation: implications for rehabilitation and recovery. Brain Stimul 7, 365–371 (2014). 10.1016/j.brs.2014.01.004 24507574

[55] Nettekoven C Inter-individual variability in cortical excitability and motor network connectivity following multiple blocks of rTMS. NeuroImage 118, 209–218 (2015). 10.1016/j.neuroimage.2015.06.004 PMC5216181 26052083

[56] Nir Y Coupling between neuronal firing rate, gamma LFP, and BOLD fMRI is related to interneuronal correlations. Curr. Biol 17, 1275–1285 (2007). 10.1016/j.cub.2007.06.066 17686438

[57] Chang C, Liu Z, Chen MC, Liu X & Duyn JH EEG correlates of time-varying BOLD functional connectivity. NeuroImage 72, 227–236 (2013). 10.1016/j.neuroimage.2013.01.049 PMC3602157 23376790

[58] Tagliazucchi E, von Wegner F, Morzelewski A, Brodbeck V & Laufs H Dynamic BOLD functional connectivity in humans and its electrophysiological correlates. Front. Hum. Neurosci 6, 339 (2012). 10.3389/fnhum.2012.00339 PMC3531919 23293596

[59] Leuchter AF, Hunter AM, Krantz DE & Cook IA Rhythms and blues: modulation of oscillatory synchrony and the mechanism of action of antidepressant treatments. Ann. N. Y. Acad. Sci 1344, 78–91 (2015). 10.1111/nyas.12742 PMC4412810 25809789

[60] Leuchter AF, Cook IA, Jin Y & Phillips B The relationship between brain oscillatory activity and therapeutic effectiveness of transcranial magnetic stimulation in the treatment of major depressive disorder. Front. Hum. Neurosci 7, 37 (2013). 10.3389/fnhum.2013.00037 PMC3581824 23550274

[61] Leuchter AF, Wilson AC, Vince-Cruz N & Corlier J Novel method for identification of individualized resonant frequencies for treatment of major depressive disorder (MDD) using repetitive transcranial magnetic stimulation (rTMS): a proof-of-concept study. Brain Stimul 14, 1373–1383 (2021). 10.1016/j.brs.2021.08.011 34425244

[62] Shine JM Distinct patterns of temporal and directional connectivity among intrinsic networks in the human brain. J. Neurosci 37, 9667–9674 (2017). 10.1523/JNEUROSCI.1574-17.2017 PMC6596608 28893929

[63] Sridharan D, Levitin DJ & Menon V A critical role for the right fronto-insular cortex in switching between central-executive and default-mode networks. Proc. Natl Acad. Sci. USA 105, 12569–12574 (2008). 10.1073/pnas.0800005105 PMC2527952 18723676

[64] Williams LM Defining biotypes for depression and anxiety based on large-scale circuit dysfunction: a theoretical review of the evidence and future directions for clinical translation. Depress. Anxiety 34, 9–24 (2017). 10.1002/da.22556 PMC5702265 27653321

[65] Zhang J Disrupted brain connectivity networks in drugnaive, first-episode major depressive disorder. Biol. Psychiatry 70, 334–342 (2011). 10.1016/j.biopsych.2011.05.018 21791259

[66] Li B A treatment-resistant default mode subnetwork in major depression. Biol. Psychiatry 74, 48–54 (2013). 10.1016/j.biopsych.2012.11.007 23273724

[67] Dubin MJ, Liston C, Avissar MA, Ilieva I & Gunning FM Network-guided transcranial magnetic stimulation for depression. Curr. Behav. Neurosci. Rep 4, 70–77 (2017). 10.1007/s40473-017-0108-7 PMC5351807 28316903

[68] Kaiser RH Abnormal frontoinsular-default network dynamics in adolescent depression and rumination: a preliminary resting-state co-activation pattern analysis. Neuropsychopharmacology 44, 1604–1612 (2019). 10.1038/s41386-019-0399-3 PMC6784913 31035283

[69] Manoliu A Insular dysfunction within the salience network is associated with severity of symptoms and aberrant inter-network connectivity in major depressive disorder. Front. Hum. Neurosci 7, 930 (2013). 10.3389/fnhum.2013.00930 PMC3896989 24478665

[70] Whitton AE Pretreatment rostral anterior cingulate cortex connectivity with salience network predicts depression recovery: findings from the EMBARC randomized clinical trial. Biol. Psychiatry 85, 872–880 (2019). 10.1016/j.biopsych.2018.12.007 PMC6499696 30718038

[71] Taylor SF Changes in brain connectivity during a sham-controlled, transcranial magnetic stimulation trial for depression. J Affect. Disord 232, 143–151 (2018). 10.1016/j.jad.2018.02.019 PMC5858982 29494898

[72] Passow S Default-mode network functional connectivity is closely related to metabolic activity. Hum. Brain Mapp 36, 2027–2038 (2015). 10.1002/hbm.22753 PMC5006878 25644693

[73] Tomasi D, Wang GJ & Volkow ND Energetic cost of brain functional connectivity. Proc. Natl Acad. Sci. USA 110, 13642–13647 (2013). 10.1073/pnas.1303346110 PMC3746878 23898179

[74] Riedl V Local activity determines functional connectivity in the resting human brain: a simultaneous FDG-PET/fMRI study. J. Neurosci 34, 6260–6266 (2014). 10.1523/JNEUROSCI.0492-14.2014 PMC6608106 24790196

[75] Rosenthal ZP Local perturbations of cortical excitability propagate differentially through large-scale functional networks. Cereb. Cortex 30, 3352–3369 (2020). 10.1093/cercor/bhz314 PMC7305790 32043145

[76] van den Heuvel MP Multimodal analysis of cortical chemoarchitecture and macroscale fMRI resting-state functional connectivity. Hum. Brain Mapp 37, 3103–3113 (2016). 10.1002/hbm.23229 PMC5111767 27207489

[77] Uddin LQ Salience processing and insular cortical function and dysfunction. Nat. Rev. Neurosci 16, 55–61 (2015). 10.1038/nrn3857 25406711

[78] Martucci KT & Mackey SC Neuroimaging of pain: human evidence and clinical relevance of central nervous system processes and modulation. Anesthesiology 128, 1241–1254 (2018). 10.1097/ALN.0000000000002137 PMC5953782 29494401

[79] Duecker F & Sack AT Rethinking the role of sham TMS. Front. Psychol 6, 210 (2015). 10.3389/fpsyg.2015.00210 PMC4341423 25767458

[80] Schmidt ME Cerebral glucose metabolism during pharmacologic studies: test-retest under placebo conditions. J. Nucl. Med 37, 1142–1149 (1996). 8965185

[81] Schaefer SM Six-month test-retest reliability of MRIdefined PET measures of regional cerebral glucose metabolic rate in selected subcortical structures. Hum. Brain Mapp 10, 1–9 (2000). 10.1002/(SICI)1097-0193(200005)10:1<1::AID-HBM10>3.0.CO;2-O PMC6871851 10843513

[82] Deng ZD, Lisanby SH & Peterchev AV Electric field depthfocality tradeoff in transcranial magnetic stimulation: simulation comparison of 50 coil designs. Brain Stimul 6, 1–13 (2013). 10.1016/j.brs.2012.02.005 PMC3568257 22483681

[83] Blumberger DM Effectiveness of theta burst versus high-frequency repetitive transcranial magnetic stimulation in patients with depression (THREE-D): a randomised non-inferiority trial. Lancet 391, 1683–1692 (2018). 10.1016/S0140-6736(18)30295-2 29726344

[84] Siebner HR Patients with focal arm dystonia have increased sensitivity to slow-frequency repetitive TMS of the dorsal premotor cortex. Brain 126, 2710–2725 (2003). 10.1093/brain/awg282 12937071

[85] Alexander L Over-activation of primate subgenual cingulate cortex enhances the cardiovascular, behavioral and neural responses to threat. Nat. Commun 11, 5386 (2020). 10.1038/s41467-020-19167-0 PMC7588412 33106488

[86] Johansen-Berg H Anatomical connectivity of the subgenual cingulate region targeted with deep brain stimulation for treatment-resistant depression. Cereb. Cortex 18, 1374–1383 (2008). 10.1093/cercor/bhm167 PMC7610815 17928332

[87] Miron JP Safety, tolerability and effectiveness of a novel 20 Hz rTMS protocol targeting dorsomedial prefrontal cortex in major depression: an open-label case series. Brain Stimul 12, 1319–1321 (2019). 10.1016/j.brs.2019.06.020 31266722

[88] Makarov SN A software toolkit for TMS electric-field modeling with boundary element fast multipole method: an efficient MATLAB implementation. J. Neural Eng 17, 046023 (2020). 10.1088/1741-2552/ab85b3 32235065

[89] Makarov SN, Noetscher GM, Raij T & Nummenmaa A A quasi-static boundary element approach with fast multipole acceleration for high-resolution bioelectromagnetic models. IEEE Trans. Biomed. Eng 65, 2675–2683 (2018). 10.1109/TBME.2018.2813261 PMC7388683 29993385

[90] Thielscher A, Antunes A & Saturnino GB Field modeling for transcranial magnetic stimulation: a useful tool to understand the physiological effects of TMS? Annu. Int. Conf. IEEE Eng. Med. Biol. Soc 2015, 222–225 (2015). 10.1109/EMBC.2015.7318340 26736240

[91] Windhoff M, Opitz A & Thielscher A Electric field calculations in brain stimulation based on finite elements: an optimized processing pipeline for the generation and usage of accurate individual head models. Hum. Brain Mapp 34, 923–935 (2013). 10.1002/hbm.21479 PMC6870291 22109746

[92] Van Dijk KR Intrinsic functional connectivity as a tool for human connectomics: theory, properties, and optimization. J. Neurophysiol 103, 297–321 (2010). 10.1152/jn.00783.2009 PMC2807224 19889849

[93] Izquierdo-Garcia D, Eldaief MC, Vangel MG & Catana C Intrascanner reproducibility of an SPM-based head MR-based attenuation correction method. IEEE Trans. Radiat. Plasma Med. Sci 3, 327–333 (2019). 10.1109/trpms.2018.2868946 PMC7291765 32537528

[94] Izquierdo-Garcia D An SPM8-based approach for attenuation correction combining segmentation and nonrigid template formation: application to simultaneous PET/MR brain imaging. J. Nucl. Med 55, 1825–1830 (2014). 10.2967/jnumed.113.136341 PMC4246705 25278515

[95] Van Dijk KR, Sabuncu MR & Buckner RL The influence of head motion on intrinsic functional connectivity MRI. NeuroImage 59, 431–438 (2012). 10.1016/j.neuroimage.2011.07.044 PMC3683830 21810475

