# Supplementary material for: Network-specific metabolic and haemodynamic effects elicited by non-invasive brain stimulation
Source: Nat Ment Health. Author manuscript; Available in PMC 2023 Nov 17. (PMC10655825; doi:10.1038/s44220-023-00046-8)
Supplement: Supplementary Material [file NIHMS1933013-supplement-Supplementary_Material.pdf]

nature mental health

Article

https://doi.org/10.1038/s44220-023-00046-8

Network-specific metabolic and haemodynamic effects elicited by non-invasive brain stimulation

In the format provided by the authors and unedited

Figure Legends

Fig. S1. Confirmation of network identity at target sites. Surface-rendered group-averaged seed-based functional connectivity (FC) maps defined using each subject's salience network (SAL) targets (*Top*, black rings) and default network (DN) targets (*Bottom*, white rings) as seeds. The SAL targets showed positive correlation with regions of the salience network (black outlines) and negative correlations with regions of the default network (white outlines). In contrast, the DN targets showed positive correlation with the default network and negative correlation with the salience network.

Fig. S2. Changes in target metabolism in each subject. Subjects are numbered in the order that data were acquired. For each subject, unthresholded images are shown displaying glucose metabolism at the target site during Baseline (*left*), following Transcranial Magnetic Stimulation (TMS) (*middle*), and the subtraction of the two (*right*). *Left panel* shows effects of SAL-TMS, with black rings representing SAL targets. *Right panel* shows effects of DN-TMS, with white rings representing DN targets.

Fig. S3. Target locations as they relate to gyral morphology. Coronal T1 images of each subject are shown in standardized MNI space. Subjects were numbered in the order in which experimental procedures were performed. The red dots represent functionally derived target sites which were targeted using a neuronavigation system during administration of TMS. The targets in each subject were far apart, with a more dorsomedial site representing the default network (DN) target, while the more inferolateral site represents the salience network (SAL) target. We did not observe systematic differences between sites, such as in the depth or cortical orientation of the target in relation to the cranial surface, that might be relevant for understanding the different effects of stimulation.

Fig. S4. Comparison of TMS induced functional connectivity (FC) between different scanning sessions. Surface rendered voxel-wise statistical *T* maps (p<0.05, FWE cluster corrected) showing effects of TMS on FC between scanning sessions (*left*, Post > Pre; *right*, Post > Baseline*)*. The top three rows represent FC changes due to stimulation of the salience network (SAL-TMS) and the bottom three rows represent FC changes due to stimulation of the default network (DN-TMS). Each row displays FC changes estimated using a different seed. 'SAL target FC' and 'DN target FC' displays regions showing differences in FC with the individually defined stimulation site in each subject, averaged across subjects. 'SAL FC' and 'DN FC' displays regions showing differences in FC with the average signal calculated from ROIs encompassing the entire default (white borders) and salience networks (black borders). For the SAL-TMS condition, between-network FC changes were more prominent in the Post > Baseline subtraction. For DN-TMS, the two comparisons differed in the magnitude of changes, but the topography and direction of FC changes were comparable, e.g., FC decreases within network and FC increases (i.e., reduced anticorrelations) between networks were observed in both comparisons.

Fig. S5. The magnitude of metabolic effects of salience network TMS correlate with FC changes across subjects. Surface rendered voxel-wise correlation (*r*) maps (p<0.05, uncorrected) depicting correlations between SAL target metabolism (WBn-suv) changes and the observed FC changes across subjects. Each row indicates FC changes from a specific seed/ROI: the SAL target itself, the sgACC and the entire SAL network as an ROI. The left panel shows regions where a positive correlation between metabolism and FC effects of SAL-TMS was observed (yellow/pink). The right panel shows regions where a negative correlation between metabolism and FC effects following SAL-TMS was observed (green/blue). Across subjects, increases in SAL target metabolism correlated with increased FC between the sgACC and the salience network, particularly frontoinsular regions of this network (*middle row*). SAL target metabolism also correlated positively with cross-network FC changes between the default and salience networks (*bottom row*).

Fig. S6. The magnitude of metabolic effects of default network TMS correlate with FC changes across subjects. Surface rendered voxel-wise correlation (*r*) maps (p<0.05, uncorrected) depicting correlations between DN target metabolism (WBn-suv) changes and the observed FC changes across subjects. Each row indicates FC changes from a specific seed/ROI: the DN target itself, the sgACC and the entire DN network as an ROI. The left panel shows regions where a positive correlation between metabolism and FC effects of DN-TMS was observed (yellow/pink). The right panel shows regions where a negative correlation between metabolism and FC effects following DN-TMS was observed (green/blue). Across subjects, decreases in DN target metabolism correlated with increased FC between the DN target and the salience network (*top row*). DN target metabolism decreases also correlated with sgACC FC decreases with the default network (*middle row*) and FC decreases within the default network proper (*bottom row*).

Fig. S7. The magnitude of transcranial magnetic stimulation (TMS) effects on metabolism at the target stimulation site is associated with the magnitude of effects of TMS on functional connectivity (FC). Surface rendered voxel-wise statistical *T* maps (p<0.05, uncorrected) showing Post > Pre TMS FC changes. The right column shows the group mean (all subjects, n=16), and the other columns show the 5 subjects with the least and most robust changes in target metabolism. The top row shows FC changes with respect to an ROI encompassing the entire salience network (SAL ROI) following SAL-TMS and the bottom row shows FC changes with respect to an ROI encompassing the entire default network (DN ROI) following DN-TMS.

Fig. S8. E-field modeling for each subject. Subjects are numbered, e.g., S1, S2. For each subject, the modeled E-field is shown in pairs for the SAL-TMS condition (*left*) and for the DN-TMS condition (*right*). Modeling was done based on tracking data from the Nexstim™ neuronavigation system, which recorded coil position/orientation during TMS for each subject (except for Subject 12, as tracking data was corrupted on the neuronavigation computer) using a Magventure™ MagPro B-65 coil (or a Magventure™ MagPro MCF-65 coil in the case of subject 7, S7) tangential to the scalp. The stimulated targets are displayed on the surface meshes as black rings for the SAL-TMS condition and white rings for the DN-TMS condition. The non-stimulated targets (e.g., the subject's DN-TMS target during SAL-TMS) are displayed as triangles. Subject doses (% maximum stimulation output, MSO) are also shown. The approximate coil position is shown on a standard head model behind the surface meshes.

Fig. S9. Replication of results using different cortical parcellation schemes. Three different parcellation schemes: Glasser et al. 2016, Gordon et al. 2016 and Power et al. 2011 are shown in the left column, in addition to the Yeo et al. 2011 parcellation used in the main analyses. DN and SAL stimulation targets are shown using black and white rings (*left* column, see **Fig 2**, main text). Each parcellation scheme uses a different name for a similar set of regions, i.e., the salience network (SAL), the cingulo-opercular network (CO), and the "task-positive" network. Right columns display the overlap between the selected network (black outlines) and regions showing metabolic increases, FC increases, and increased correlation between target metabolism and FC changes following the two transcranial magnetic stimulation (TMS) conditions (SAL-TMS and DN-TMS). This demonstrates that these selected results engage the same network regardless of the parcellation scheme used. DN=default network, SAL=salience network, CO=cingulo-opercular network, FPN=frontoparietal network.

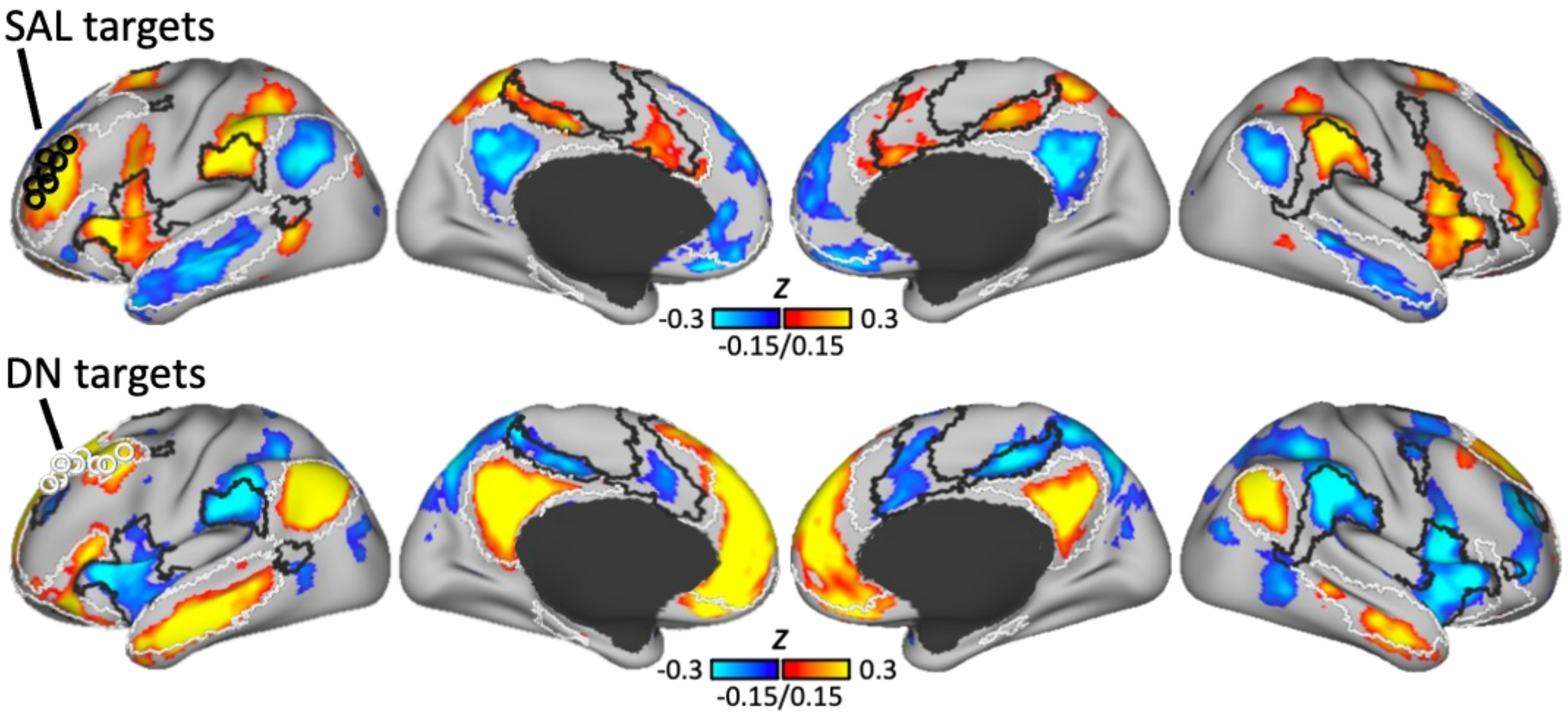
SAL targets
Z
-0.3
0.3
-0.15/0.15
DN targets
Z
-0.3
0.3
-0.15/0.15

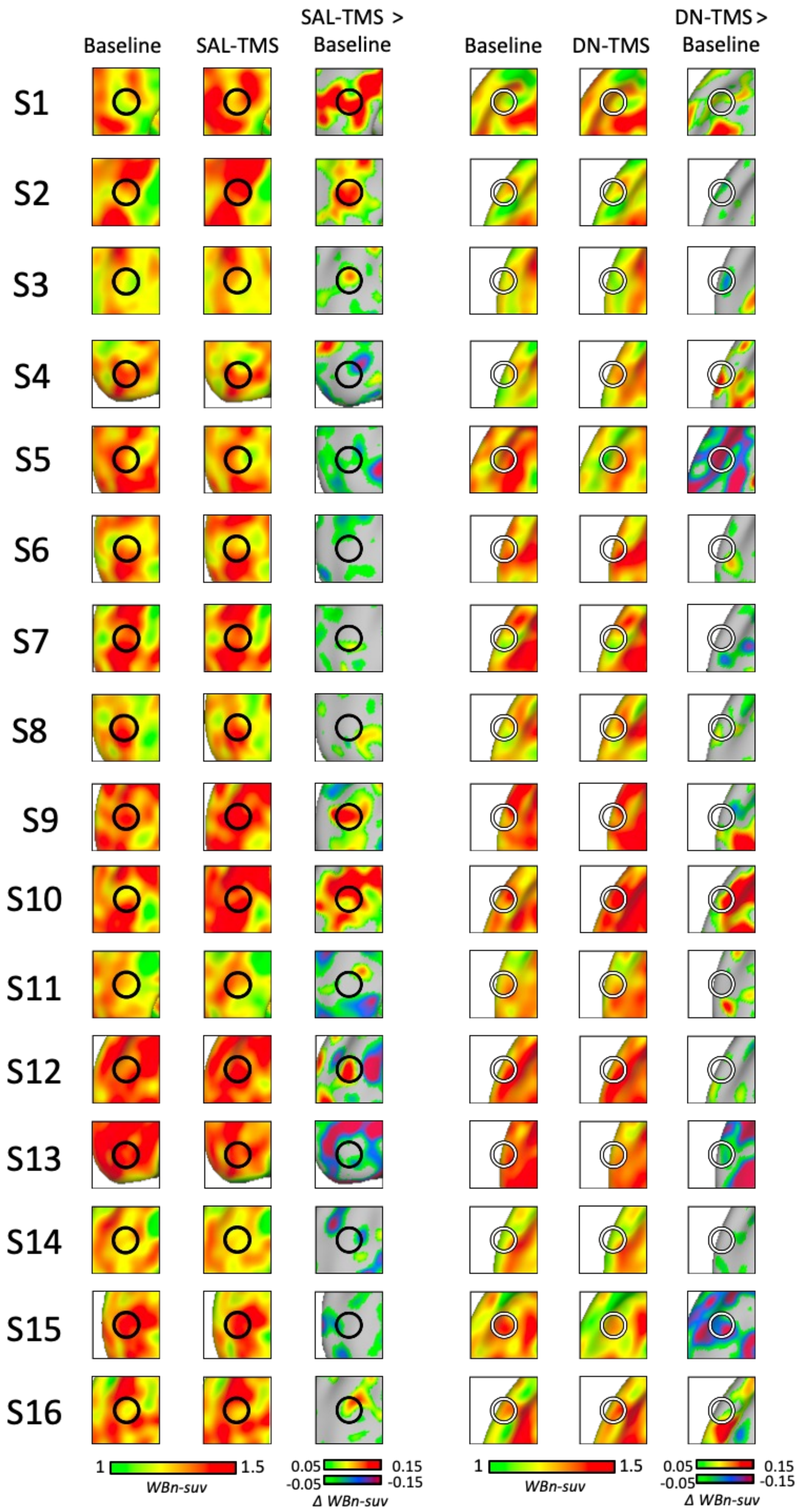

Baseline
SAL-TMS
SAL-TMS > Baseline
Baseline
DN-TMS
DN-TMS > Baseline
S1
S2
S3
S4
S5
S6
S7
S8
S9
S10
S11
S12
S13
S14
S15
S16
1
1.5
WBn-suv
0.05
0.15
-0.05
-0.15
Δ WBn-suv

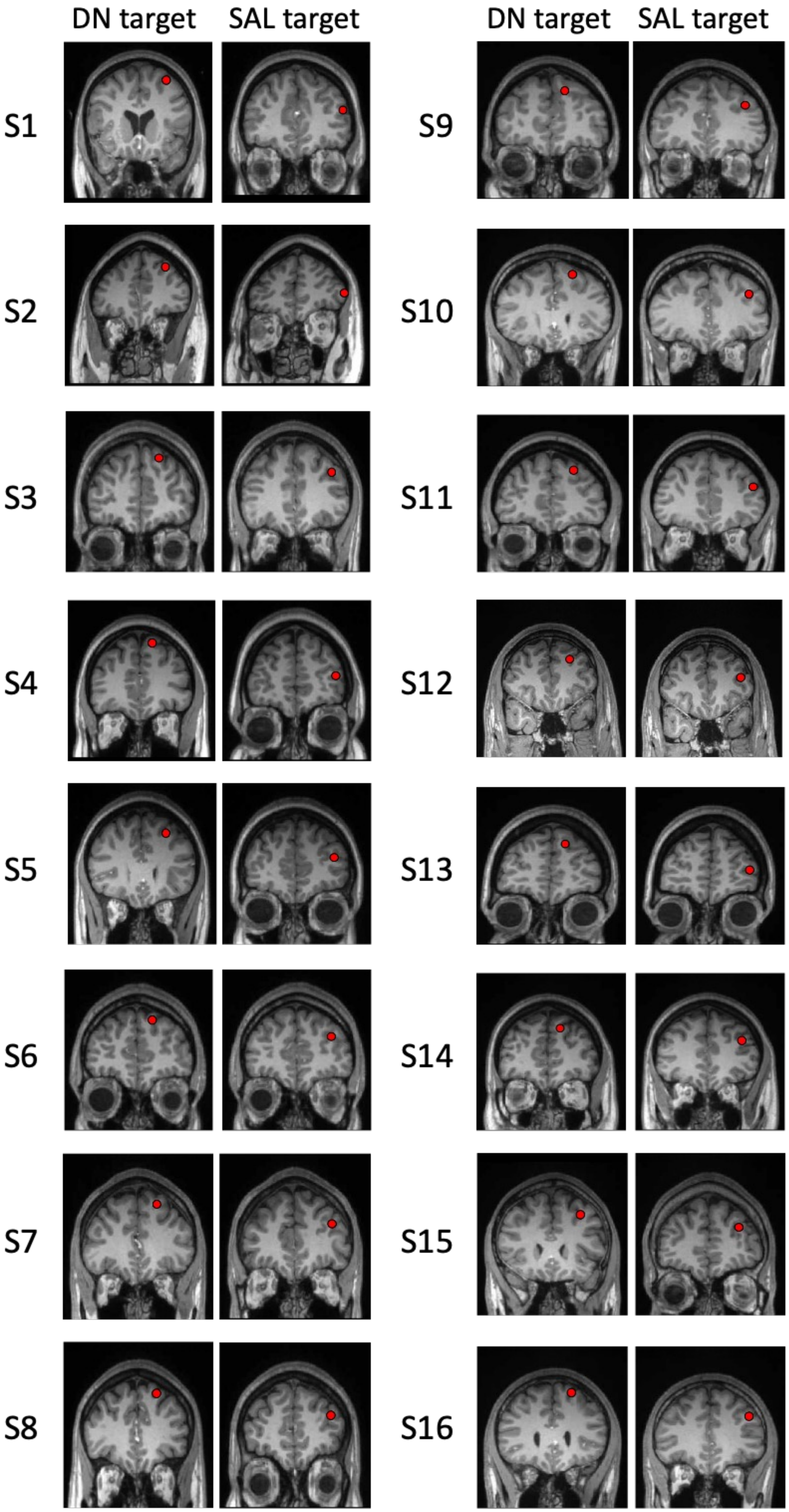
DN target
SAL target
DN target
SAL target
S1
S2
S3
S4
S5
S6
S7
S8
S9
S10
S11
S12
S13
S14
S15
S16

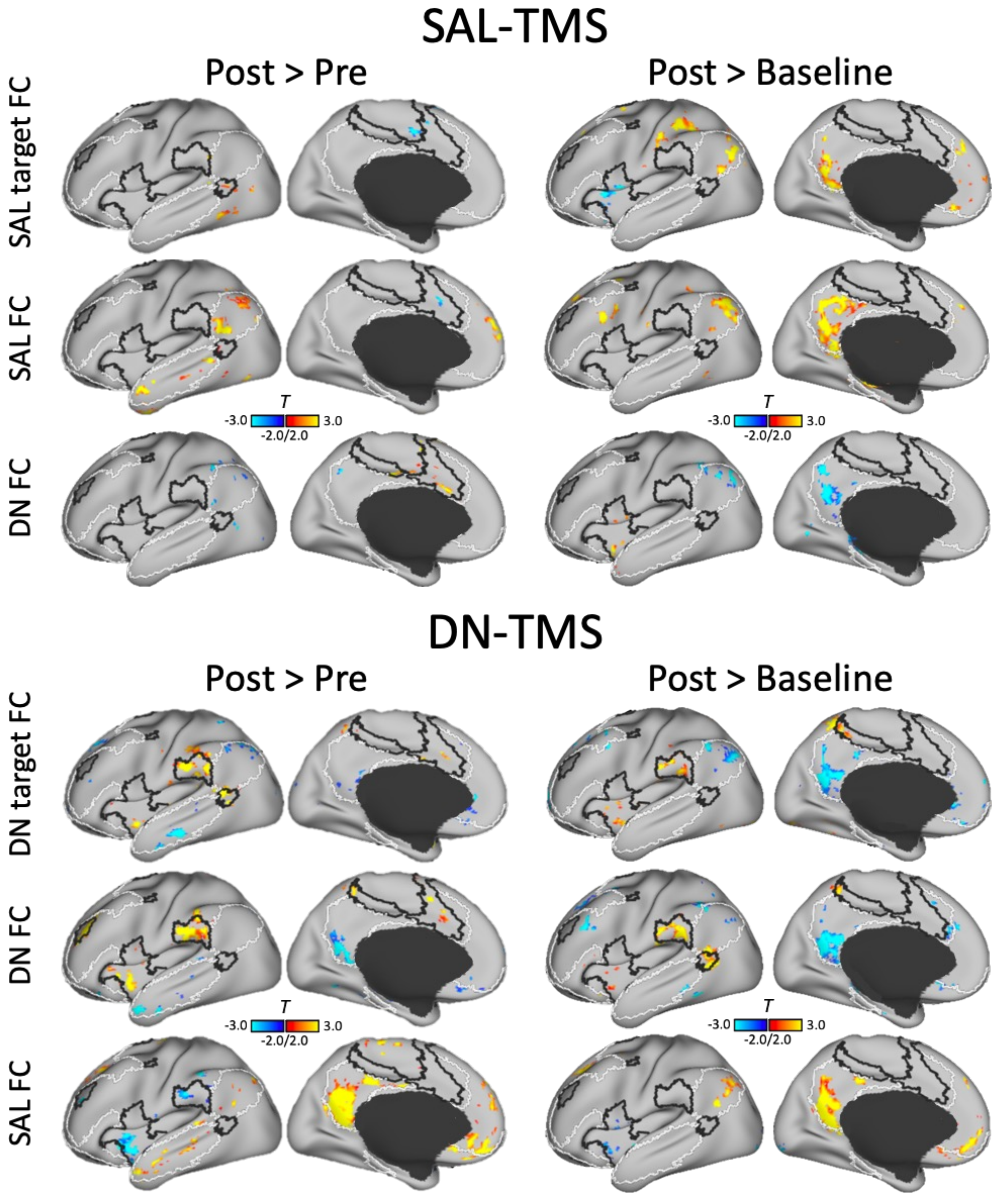
SAL-TMS
Post > Pre
Post > Baseline
SAL target FC
SAL FC
DN FC
T
-3.0
3.0
-2.0/2.0
DN-TMS
Post > Pre
Post > Baseline
DN target FC
DN FC
SAL FC

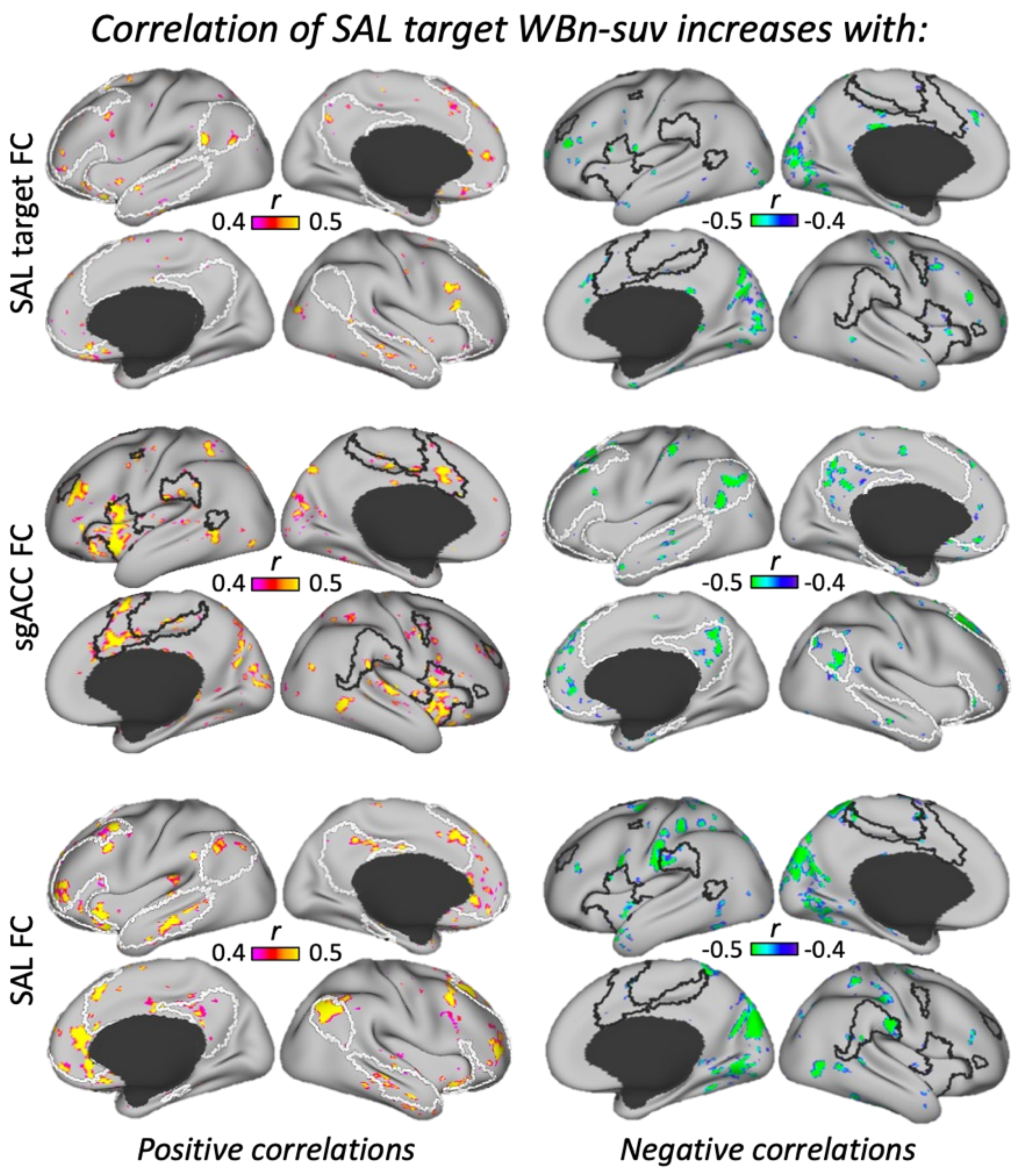
Correlation of SAL target WBn-suv increases with:
SAL target FC
sgACC FC
SAL FC
r
0.4
0.5
-0.5
-0.4
Positive correlations
Negative correlations

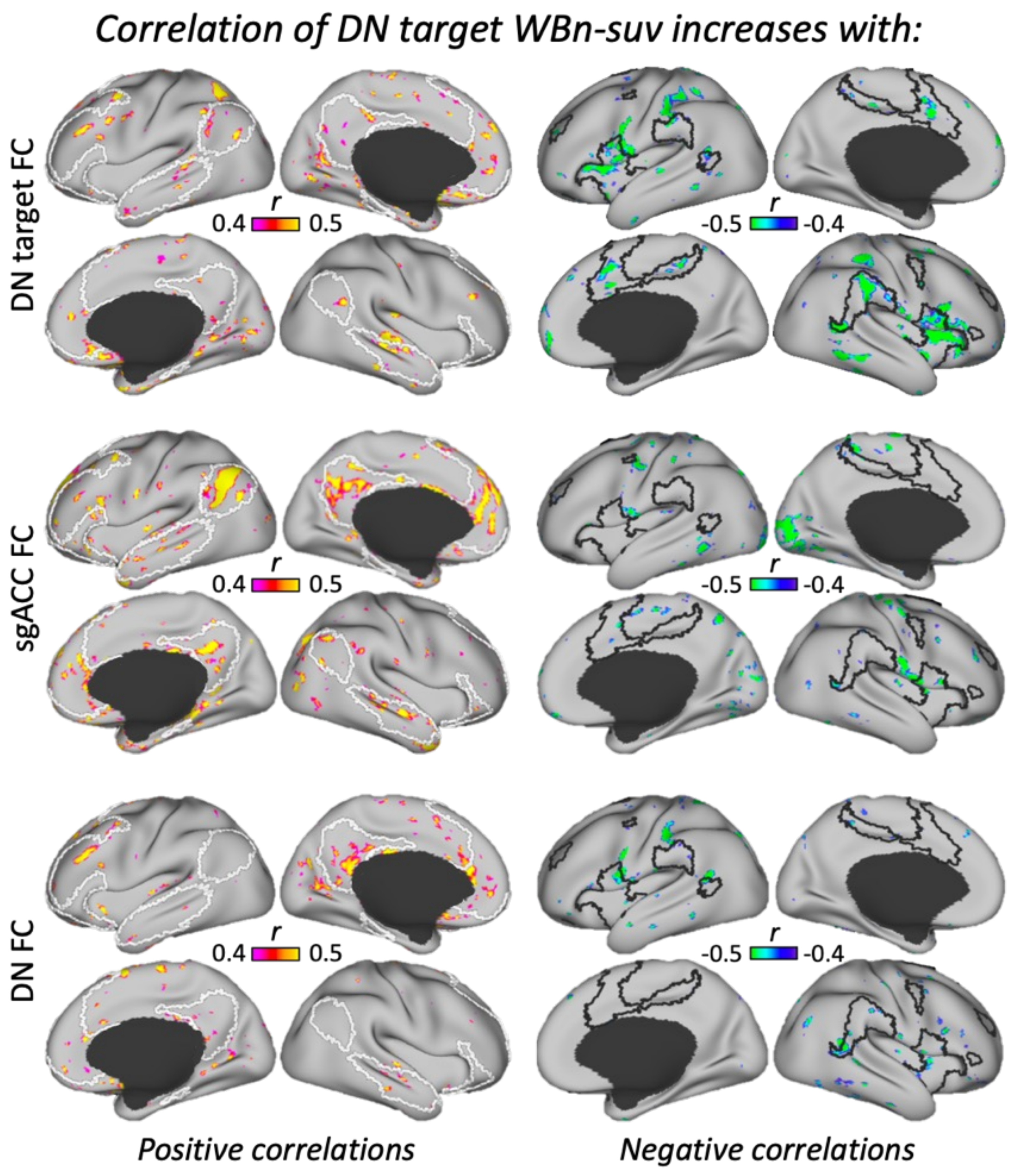
Correlation of DN target WBn-suv increases with:
DN target FC
sgACC FC
DN FC
r
0.4
0.5
-0.5
-0.4
Positive correlations
Negative correlations

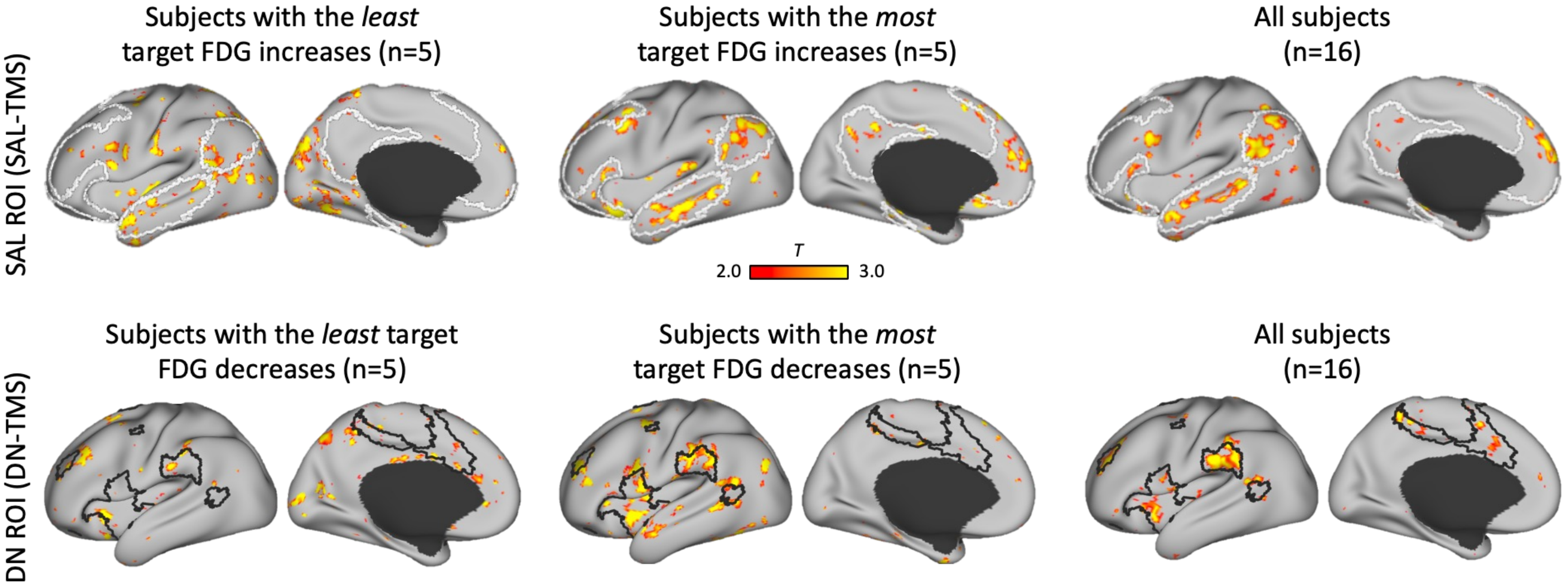
SAL ROI (SAL-TMS)
Subjects with the *least* target FDG increases (n=5)
Subjects with the *most* target FDG increases (n=5)
All subjects (n=16)
T
2.0
3.0
DN ROI (DN-TMS)
Subjects with the *least* target FDG decreases (n=5)
Subjects with the *most* target FDG decreases (n=5)
All subjects (n=16)

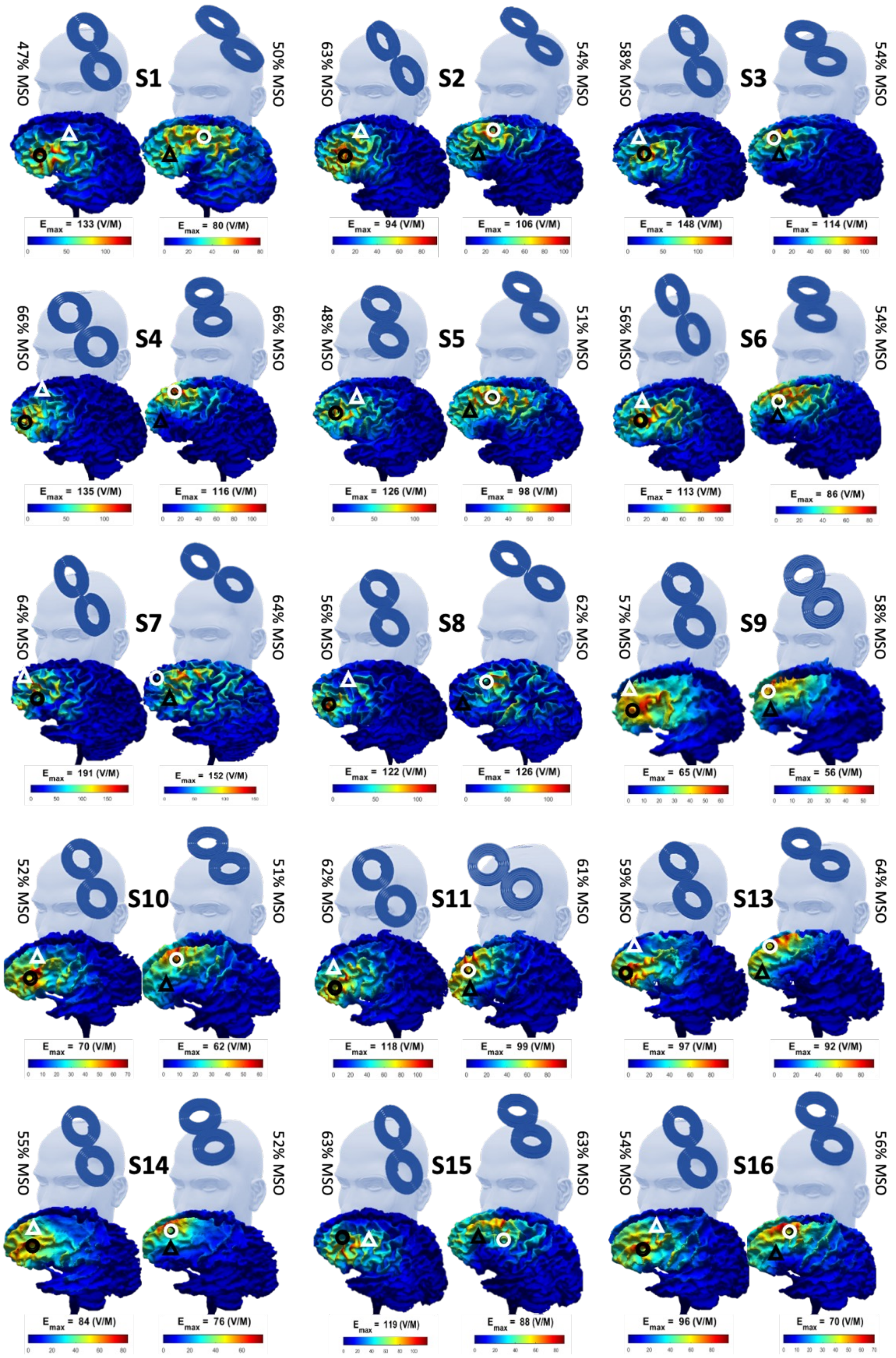
47% MSO
S1
50% MSO
Emax = 133 (V/M)
Emax = 80 (V/M)
63% MSO
S2
54% MSO
Emax = 94 (V/M)
Emax = 106 (V/M)
58% MSO
S3
54% MSO
Emax = 148 (V/M)
Emax = 114 (V/M)
66% MSO
S4
66% MSO
Emax = 135 (V/M)
Emax = 116 (V/M)
48% MSO
S5
51% MSO
Emax = 126 (V/M)
Emax = 98 (V/M)
56% MSO
S6
54% MSO
Emax = 113 (V/M)
Emax = 86 (V/M)
64% MSO
S7
64% MSO
Emax = 191 (V/M)
Emax = 152 (V/M)
56% MSO
S8
62% MSO
Emax = 122 (V/M)
Emax = 126 (V/M)
57% MSO
S9
58% MSO
Emax = 65 (V/M)
Emax = 56 (V/M)
52% MSO
S10
51% MSO
Emax = 70 (V/M)
Emax = 62 (V/M)
62% MSO
S11
61% MSO
Emax = 118 (V/M)
Emax = 99 (V/M)
59% MSO
S13
64% MSO
Emax = 97 (V/M)
Emax = 92 (V/M)
55% MSO
S14
52% MSO
Emax = 84 (V/M)
Emax = 76 (V/M)
63% MSO
S15
63% MSO
Emax = 119 (V/M)
Emax = 88 (V/M)
54% MSO
S16
56% MSO
Emax = 96 (V/M)
Emax = 70 (V/M)

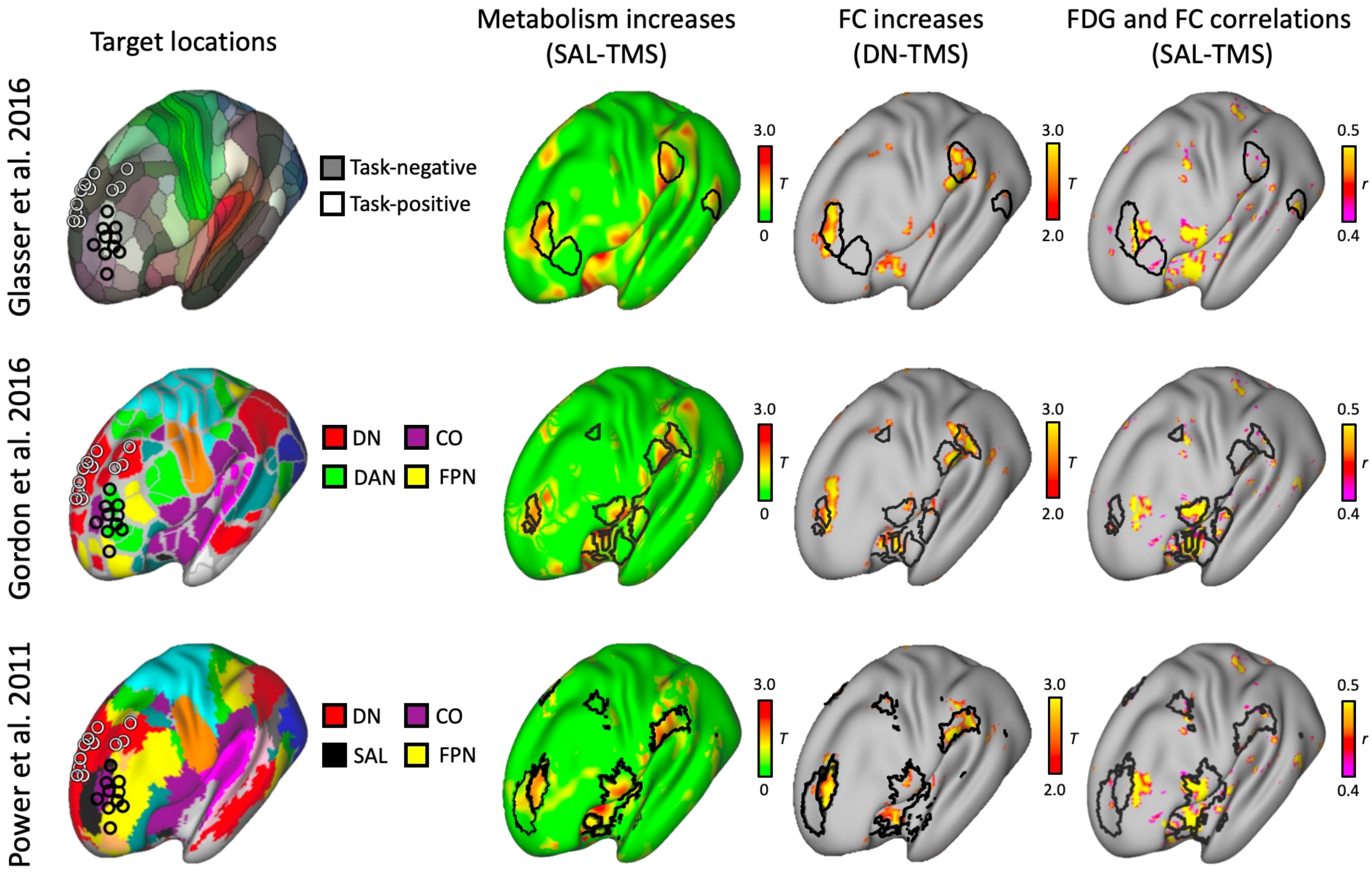

Target locations
Metabolism increases
(SAL-TMS)
FC increases
(DN-TMS)
FDG and FC correlations
(SAL-TMS)
Glasser et al. 2016
Task-negative
Task-positive
Gordon et al. 2016
DN
CO
DAN
FPN
Power et al. 2011
DN
CO
SAL
FPN
3.0
T
0
2.0
0.5
r
0.4
